# Comparative Effects of Metamizole (Dipyrone) and Naproxen on Renal Function and Prostacyclin Synthesis in Salt-Depleted Healthy Subjects - A Randomized Controlled Parallel Group Study

**DOI:** 10.3389/fphar.2021.620635

**Published:** 2021-09-07

**Authors:** Lea S Blaser, Urs Duthaler, Jamal Bouitbir, Anne B Leuppi-Taegtmeyer, Evangelia Liakoni, Reto Dolf, Michael Mayr, Jürgen Drewe, Stephan Krähenbühl, Manuel Haschke

**Affiliations:** ^1^ Division of Clinical Pharmacology & Toxicology, University Hospital Basel, Basel, Switzerland; ^2^ Department of Biomedicine, University of Basel, Basel, Switzerland; ^3^ Department of Clinical Research, University of Basel, Basel, Switzerland; ^4^ Clinical Pharmacology and Toxicology, Department of General Internal Medicine, Inselspital, Bern University Hospital, University of Bern, Bern, Switzerland; ^5^ Office of Environment and Energy, Basel, Switzerland; ^6^ Medical Outpatient Department, University Hospital Basel, Basel, Switzerland

**Keywords:** metamizole, 4-methylaminoantipyrine (4-MAA), inulin clearance, urinary sodium excretion, prostacyclin, 6-keto-PGF1α, salt-depleted healthy volunteers

## Abstract

**Aim:** The objective was to investigate the effect of metamizole on renal function in healthy, salt-depleted volunteers. In addition, the pharmacokinetics of the four major metamizole metabolites were assessed and correlated with the pharmacodynamic effect using urinary excretion of the prostacyclin metabolite 6-keto-prostaglandin F1α.

**Methods:** Fifteen healthy male volunteers were studied in an open-label randomized controlled parallel group study. Eight subjects received oral metamizole 1,000 mg three times daily and seven subjects naproxen 500 mg twice daily for 7 days. All subjects were on a low sodium diet (50 mmol sodium/day) starting 1 week prior to dosing until the end of the study. Glomerular filtration rate was measured using inulin clearance. Urinary excretion of sodium, potassium, creatinine, 6-keto-prostaglandin F1α, and pharmacokinetic parameters of naproxen and metamizole metabolites were assessed after the first and after repeated dosing.

**Results:** In moderately sodium-depleted healthy subjects, single or multiple dose metamizole or naproxen did not significantly affect inulin and creatinine clearance or sodium excretion. Both drugs reduced renal 6-keto-prostaglandin F1α excretion after single and repeated dosing. The effect started 2 h after intake, persisted for the entire dosing period and correlated with the concentration-profile of naproxen and the active metamizole metabolite 4-methylaminoantipyrine (4-MAA). PKPD modelling indicated less potent COX-inhibition by 4-MAA (EC_50_ 0.69 ± 0.27 µM) compared with naproxen (EC_50_ 0.034 ± 0.033 µM).

**Conclusions:** Short term treatment with metamizole or naproxen has no significant effect on renal function in moderately sodium depleted healthy subjects. At clinically relevant doses, 4-MAA and naproxen both inhibit COX-mediated renal prostacyclin synthesis.

## Introduction

**FIGURE 1:**
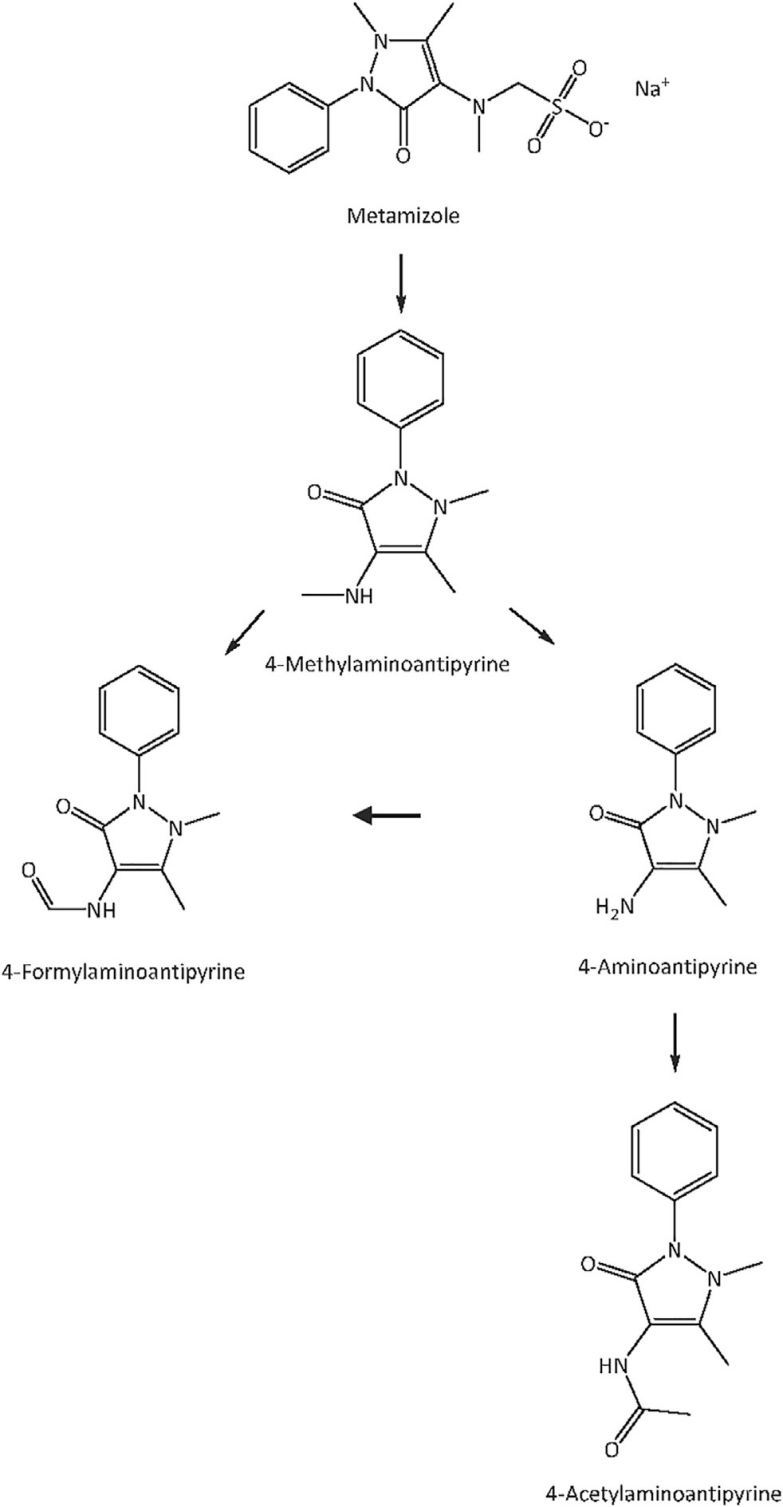
Metabolism of metamizole. The prodrug metamizole is non-enzymatically cleaved to 4-N-methylaminoantipyrine (4-MAA), which is N-demethylated to 4-aminoantipyrine (4-AA). 4-AA can be N-formylated to 4-N-formylaminoantipyrine (4-FAA) or N-acetylated to 4-N-acetylaminoantipyrine (4-AAA).

Metamizole (dipyrone) is an effective non-opioid analgesic and antipyretic drug. It has a favorable gastrointestinal safety profile, but there is a lack of data on its renal tolerability compared with classic nonsteroidal anti-inflammatory drugs (NSAIDs) (Farker et al., 1995; Zapater et al., 2015). Metamizole is a prodrug (Figure 1) and reaches the systemic circulation after non-enzymatic cleavage to 4-N-methylaminoantipyrine (4-MAA), which can be N-demethylated to 4-aminoantipyrine (4-AA). 4-AA can be N-formylated to 4-N-formylaminoantipyrine (4-FAA) or N-acetylated to 4-N-acetylaminoantipyrine (4-AAA).

The mechanism of action of metamizole is not entirely clear. Inhibition of cyclooxygenase (COX) enzymes by metamizole metabolites has been demonstrated in *ex vivo* and *in vitro* experiments. However, the reported IC_50_ values for COX-inhibition show high variability, ranging from 2.6 μmol/L to >400 μmol/L and mostly appear to be higher compared to corresponding IC_50_ values of non-selective NSAIDs such as ibuprofen (12–42 μmol/L) or naproxen (0.3–24 μmol/L) (Campos et al., 1999; Gierse et al., 1999; Hinz et al., 2007; Pierre et al., 2007; Rogosch et al., 2012). Other proposed mechanisms of action are stimulation of endogenous cannabinoid receptors (Rogosch et al., 2012; Crunfli et al., 2015), involvement of glutamatergic mechanisms, inhibition of neurokinin-1 receptor or transient receptor potential ankyrin 1(TRPA1) channel mediated responses (Nassini et al., 2015), inhibition of the protein kinase C-dependent pathway (Siebel et al., 2004), and involvement of the descending serotonergic and noradrenergic systems (Gencer et al., 2015).

Regarding safety, the most frequently discussed problem associated with metamizole is agranulocytosis, a rare but potentially life-threatening adverse drug reaction (Anonymous, 1986). In addition, metamizole can cause infusion reactions with profound hypotension, especially after rapid infusion of high doses (Leone et al., 2005). The most frequent adverse reaction caused by NSAIDs is gastrointestinal toxicity, ranging from dyspepsia to severe gastrointestinal ulcerations and bleeding (CoxibBhala et al., 2013; Scarpignato et al., 2015). Based on data from animal studies (Sánchez et al., 2002a) and case-control studies in patients (García Rodríguez and Barreales Tolosa, 2007; García Rodríguez et al., 2011; de Abajo et al., 2013; Andrade et al., 2016), metamizole has a more favorable gastrointestinal safety profile, indicating clinically less relevant COX-1 inhibition in the gastrointestinal tract compared to NSAIDs (LaporteCarne et al., 1991; Sánchez et al., 2002b; Lanas et al., 2003). In addition, NSAIDs and selective cyclooxygenase-2 (COX-2) inhibitors are associated with an increased cardiovascular risk, particularly in patients with preexisting disease (Fosbol et al., 2009; Gislason et al., 2009; CoxibBhala et al., 2013). For metamizole, so far no increased cardiovascular risk has been reported (de Abajo et al., 2014); this however may be due to insufficient study data on the cardiovascular risk of metamizole compared to NSAIDs.

The mechanism leading to impaired renal perfusion associated with NSAIDs is well established. Vasodilatory prostaglandins such as prostacyclin are crucial for maintaining sufficient renal perfusion in conditions with low intravascular pressure; inhibition of renal prostaglandin synthesis by NSAIDs can therefore compromise renal autoregulation. Salt depletion is assumed to stimulate renal counter-regulations similar to those induced by hypovolemia and to increase susceptibility of the kidneys to adverse effects of COX-inhibitors in healthy individuals (Hörl, 2010). Whether metamizole is a valuable alternative to NSAIDs for patients with reduced intravascular volume has not been studied in detail. Only sporadic case reports of acute kidney injury or acute interstitial nephritis in patients treated with metamizole are available (Berruti et al., 1998; Abu-Kishk et al., 2010; Hassan et al., 2011).

Considering the increasing use of metamizole in many countries (Stammschulte et al., 2015) and the lack of study data on its effect on renal function, the primary aim of this study was to examine the effect of metamizole compared with the non-specific COX-inhibitor naproxen on renal function in healthy, salt-depleted, male volunteers. Renal function was assessed by measuring glomerular filtration using inulin clearance as well as measuring urinary excretion of sodium, potassium, creatinine and the stable prostacyclin metabolite 6-keto-prostaglandin F1α (6-keto-PGF1α). A secondary aim was to obtain pharmacokinetic data for the four major metamizole metabolites and to correlate these data with pharmacodynamic effects.

## Methods

### Materials

Metamizole was administered as Novalgin® 500 mg tablets (Sanofi-Aventis Suisse SA, Vernier, Switzerland) and naproxen as Naproxen-Mepha® Lactabs 500 mg (Mepha Pharma AG, Basel, Switzerland). Inulin (Inutest® 25% ampules) was from Fresenius Medical Care AG (Oberdorf, Switzerland, manufactured by Fresenius Kabi Austria GmbH, Linz, Austria). d-Glucose/d-Fructose Test-Combination was from R-Biopharm AG (Darmstadt, Germany), glucose-oxidase from *Aspergillus niger* and invertase from baker’s yeast (*S. cerevisiae*) were from Sigma-Aldrich (Buchs, Switzerland). Citric acid, sodium citrate, and hydrogen peroxide 30% were also purchased from Sigma-Aldrich (Buchs, Switzerland).

Gradient grade water, methanol, isopropanol and formic acid of analysis grade (98–100% purity) for liquid chromatography were obtained from Merck (Darmstadt, Germany). Chloroform was from Carl Roth (Karlsruhe, Germany) and O-(2,3,4,5,6-pentafluorobenzyl)hydroxylamine hydrochloride (PFBHA) was from Sigma-Aldrich (Buchs, Switzerland). All reference compounds and internal standards for naproxen and metamizole quantification were products of Toronto Research Chemicals (Toronto, Canada). 6-keto PGF1α, 6-keto PGF1α-d4, and 2,3-dinor-6-keto-PGF1-α were obtained from Cayman Chemicals (Ann Arbor, Michigan, United States). Stock solutions of naproxen (10 mg/ml), 6-Keto PGF1α-d4 (1 mg/ml) and the metamizole metabolites 4-methylaminoantipyrine (4-MAA; 10 mg/ml), 4-aminoantipyrine (4-AA; 5 mg/ml), 4-formylaminoantipyrine (4-FAA; 5 mg/ml), and 4-acetylaminoantipyrine (4-AAA; 5 mg/ml) were prepared in dimethyl sulfoxide (DMSO; Sigma-Aldrich, St. Louis, United States). The internal standards 2,3-Dinor 6-Keto PGF1α-d9, 4-MAA-d3, 4-AA-d3, and 4-AAA-d3, and naproxen-d3 were dissolved in DMSO to a final concentration of 1 mg/ml.

### Study Protocol

This single-center, randomized, open-label, parallel-group study in healthy, male subjects was approved by the local ethics committee (Ethikkommission beider Basel, EKBB 264/13) and the Swiss Agency for Therapeutic Products (Swissmedic Bern, protocol number 2013DR1187). The study was conducted in the phase I research unit at the University Hospital Basel, Switzerland and was registered at ClinicalTrials.gov (NCT01995006). Before inclusion into the study, we obtained written informed consent and performed a physical examination including routine laboratory tests of each subject. Healthy male subjects, without history of gastrointestinal disease or intolerance to NSAIDs were eligible (list with complete inclusion and exclusion criteria provided as Supplementary Table S1). According to the protocol, 16 subjects were planned to be included with subsequent 1:1 randomization according to a pre-specified randomization list into two treatment groups. One group was to be treated with a single dose of 1,000 mg metamizole on day 1 followed by 1000 mg metamizole three times daily from day 2 to day 7, the other group with a single dose of 500 mg naproxen on day 1 followed by 500 mg naproxen twice daily from day 2 to day 7 days. One week before the first study day, all subjects were started on a low sodium diet containing approximately 50 mmol sodium per day (Muther et al., 1981; Rossat et al., 1999). The diet was maintained during the whole study period (i.e., until the last blood sampling on day 8). Lunch and dinner with reduced sodium content were provided at the University Hospital Basel during the whole study period. For breakfast and snacks, we provided written dietary instructions. Dietary sodium intake was monitored by measuring urinary sodium excretion in 24 h urine with sample collection starting on days -4, 1, and 7 to assess compliance with the low sodium diet (Supplementary Figure S1, Table S2). Plasma samples for the pharmacokinetic evaluation were drawn before, and 1, 2, 3, 4, 5, 8, 12, and 24 h after drug administration. Starting on day 2, the study drugs were given at the regular dosing interval until day 7. On day 7, the PK measurements were repeated at the same time-points as on day 1.

### Measurement of Inulin Clearance

On day 1, after an overnight fast, a venous catheter was placed on each forearm. After the collection of baseline plasma samples, we started an inulin infusion at 125 mg/min for 20 min followed by 28 mg/min for 400 min according to the instructions of the manufacturer. After an equilibration period of 1 hour, we collected a urine sample to measure baseline inulin concentrations (t_-1_ to t_0_), before subjects ingested a single dose of either 1,000 mg metamizole or 500 mg naproxen (t_0_). We continued to collect hourly urine and plasma samples for inulin concentration measurements for the next 5 hours (t_1_ - t_5_). Inulin clearance was calculated according the following formula:

Clearanceinulin=inulinurine×volumeurine×1.73inulinplasma×time×bs bs= size (cm)*weight(kg)3600

where *inulin*_*urine*_ is the inulin concentration in urine, *volume*_*urine*_ the volume of urine, *inulin*_*plasma*_ the inulin concentration in plasma, *time* the collecting period, and *bs* the body surface. To ensure a minimal urine flow of 2–3 ml/min (according to the manufacturer’s recommendation), we instructed the study participants to drink 700 ml of water within the first 30 min after the start of the inulin infusion, followed by additional 300 ml of water per hour until the end of the inulin infusion (t_5_).

Inulin concentrations in plasma and urine were measured using an enzymatic method without deproteinization as described previously (Kuehnle et al., 1992). Standard curves were linear in plasma (mean *r* ^2^ = 0.9994, *n* = 17) and in urine (mean *r* ^2^ = 0.9991, *n* = 12). For different concentration calibrators, the coefficients of variation for repetitive measurements were 6.2–9.6% for plasma and 9.6–11.2% for urine.

### Creatinine Renal Excretion and Clearance

We determined the urinary creatinine excretion by multiplying the urine volume by the corresponding urine creatinine concentration. Division of the urinary creatinine excretion per time by the corresponding creatinine serum concentration yielded the creatinine clearance after normalization to 1.73 m^2^ body surface.

### Quantification of Sodium and Potassium Excretion in Urine

We measured sodium and potassium excretion in aliquots of hourly urine collections. Potassium concentrations in urine were analyzed using ion-specific electrodes at the central laboratory of the University Hospital Basel. Sodium concentrations in urine were analyzed at the environmental health and safety laboratory, Office of Environment and Energy of the Canton of Basel, Switzerland, using an inductively coupled plasma mass spectrometry (ICP-MS) method (DIN EN ISO 17294-2:2005-02) with a detection limit of 0.05 mg/L.

### Quantification of Naproxen and Metamizole Metabolites

Concentrations of naproxen and metamizole metabolites were measured on a Shimadzu HPLC system (Kyoto, Japan) coupled to an API 4000 tandem mass spectrometer (AB Sciex, Massachusetts, United States). The HPLC system consisted of two LC-20AD pumps, two LC-20AD XR pumps, a CTO-20AC column oven, a DGU-20A5 degassing unit, a CBM-20A controller, and a CTC HTS PAL autosampler (Zwingen, Switzerland). The LC-MS/MS system was operated using Analyst software version 1.6.1 (AB Sciex, MA, United States).

4-MAA, 4-AA, 4-FAA, and 4-AAA were separated on a pentafluorophenyl phase column (Luna 3 µ PFP 50 mm × 2.0 mm, Phenomenex, CA, United States) using water (A) and methanol (B), both supplemented with 0.1% (v/v) formic acid as mobile phases. The following mobile phase gradient program was used: 0–0.25 min; 2% mobile B, 0.25–3.5 min; 2–55% mobile B, 3.51–4.5; 95% mobile B, 4.51–5.0 min; 2% mobile B. The retention times of 4-MAA, 4-AA, 4-FAA and 4-AAA were 1.62, 1.74, 2.43, and 2.5 min, respectively. For chromatographic separation the column was kept at 43°C. All analytes were detected in positive mode using electrospray ionization.

Naproxen was eluted on a reversed phase core-shell C18 column (Kinetex 2.6 µm, 50 mm × 2.1 mm; Phenomenex, CA, United States). Similar mobile phases were used as for the metamizole analysis. The HPLC gradient program was: 0–0.25 min; 40% mobile B, 0.25–1 min; 40–95% mobile B, 1–2 min; 95% mobile B, 2.01–2.25 min; 40% mobile B. Naproxen eluted after 1.3 min. Naproxen and naproxen-d3 were detected in negative ionization mode. General settings of the mass spectrometer were similar to the ones used for the analysis of metamizole except the ion spray voltage which was set to −4500 V.

Calibration lines were prepared in drug free blank human plasma. Analyte stock solutions were serially diluted with DMSO and each dilution was mixed with blank plasma at a ratio of 1:100. Calibration ranges which included 10 or 11 calibrators were established from 25–50′000 ng/ml for naproxen, from 50–50′000 ng/ml for 4-MAA, from 25–5,000 ng/ml for 4-FAA, and from 5–5,000 ng/ml for 4-AA and 4-AAA. A correlation coefficient (*R* ^2^) of at least 0.99 was required. Quality control samples were prepared at low, medium, and high concentration levels. A mean accuracy of 85–115% (80–120% at the lower limit of quantification (LLOQ)) and a precision of 15% (LLOQ: 20%) was accepted in our study.

Plasma aliquots of 50 µl were precipitated with 500 µl methanol containing the internal standards (4-AA-d3: 10 ng/ml, 4-AAA-d3: 50 ng/ml, 4-MAA-d3: 25 ng/ml, naproxen-d3: 500 ng/ml). Samples were mixed for 1 min and centrifuged at 3,220 g for 30 min (Eppendorf 5810R, Hamburg, Germany). 2 µl supernatant were injected into the LC-MS/MS system.

### Quantification of Urinary 6-Keto-PGF1-α

Concentrations of 6-keto-PGF1- *α* in urine were measured on a UPLC system from Shimadzu connected to an AB Sciex API 5500 tandem mass spectrometer. The UPLC system had the same configuration as the HPLC system used for naproxen and metamizole except that four 30-AD pumps and a SIL-30AC autosampler were used.

Derivatization of the ketone group of 6-keto PGF1α using PFBHA resulted in the formation of two isomers. These isomers were separated on a Kinetex C18 column (2.6 µm, 50 mm × 2.1 mm, Phenomenex, CA, United States). Mobile phase A was a 5 mM ammonium bicarbonate (Sigma-Aldrich, MO, United States) solution in water, for mobile phase B isopropanol was used. The introduced samples (50 µl) were pre-column diluted *via* a T-union during the first 0.5 min of each run with mobile phase A. The following mobile phase B gradient program was used: 0–11 min; 2–23%, 11–12 min; 23–95%, 12–13 min; 95%, 13–13.5 min; 2%. 2,3-dinor-6-keto-PGF1α eluted after 9.4 min and the two PFBHA-6-keto PGF1α isomers, had a retention time of 10.0 and 10.4 min, respectively. All analytes were detected in negative mode using atmospheric pressure chemical ionization. The applied mass transitions and compound specific parameters are shown in Supplementary Table S3.

Calibration lines with 10 calibrators from 10 to 2,500 pg/ml were prepared by spiking blank human urine with 6-keto PGF1α-d4 (in DMSO) at a ratio of 1:1,000. Quality control samples were prepared at low (75 pg/ml), medium (250 pg/ml), and high (750 pg/ml) concentration levels. We applied a modified liquid-liquid extraction technique as described by Sterz et al. (Sterz et al., 2012). Aliquots of 2 ml urine were used for analysis. Before extraction, 20 μl of acetic acid were added to each sample, followed by 8 ml of the IS (2,3-dinor-6-keto-PGF1α) in a methanol-chloroform (2:1 v/v) mixture. The samples were mixed during half an hour and incubated for another half an hour at room temperature. After adding chloroform and water (2.5 ml each), the samples were mixed for 10 min and afterwards centrifuged for 10 min at 800 g. The chloroform phase was evaporated under a gentle stream of nitrogen at 40°C with a Turbo Vap LV (Caliper Life Science, MA, United States). The residue was dissolved in a 200 μl isopropanol-water-PFBHA solution (4:5:1, v/v) and incubated for 30 min at 60°C for derivatization (Blatnik and Steenwyk, 2010). 50 µl was injected into the LC-MS/MS system. Quantified concentrations of 6-keto-PGF1α were normalized to the creatinine content of the urine samples to compensate for differences in urine dilution. Values below the LLOQ of the analytical method were set to the LLOQ value.

### Pharmacokinetic Analysis

Plasma concentration data were analyzed using non-compartmental methods. Peak plasma concentrations (C_max_) and time to reach C_max_ (t_max_) were directly obtained from observed concentration-time data. The terminal elimination rate constant (λ_z_) was obtained by log-linear regression using at least three data points in the elimination phase. The area under the concentration-time curve (AUC) from zero to infinity after dosing (AUC_0-inf_obs_) was estimated using the linear trapezoidal method and was extrapolated to infinity based on the last observed or predicted concentration. Partial area under concentration-time curve from 0 to 5 h (AUC_0-5h_) was calculated for comparison of the two treatment days. The terminal elimination half-life was calculated using λ_z_. Calculations were done using the PK Solver add-in (version 2.0) for Microsoft Excel (Zhang et al., 2010).

### Pharmacokinetic and Pharmacodynamic Modeling

**FIGURE 2:**
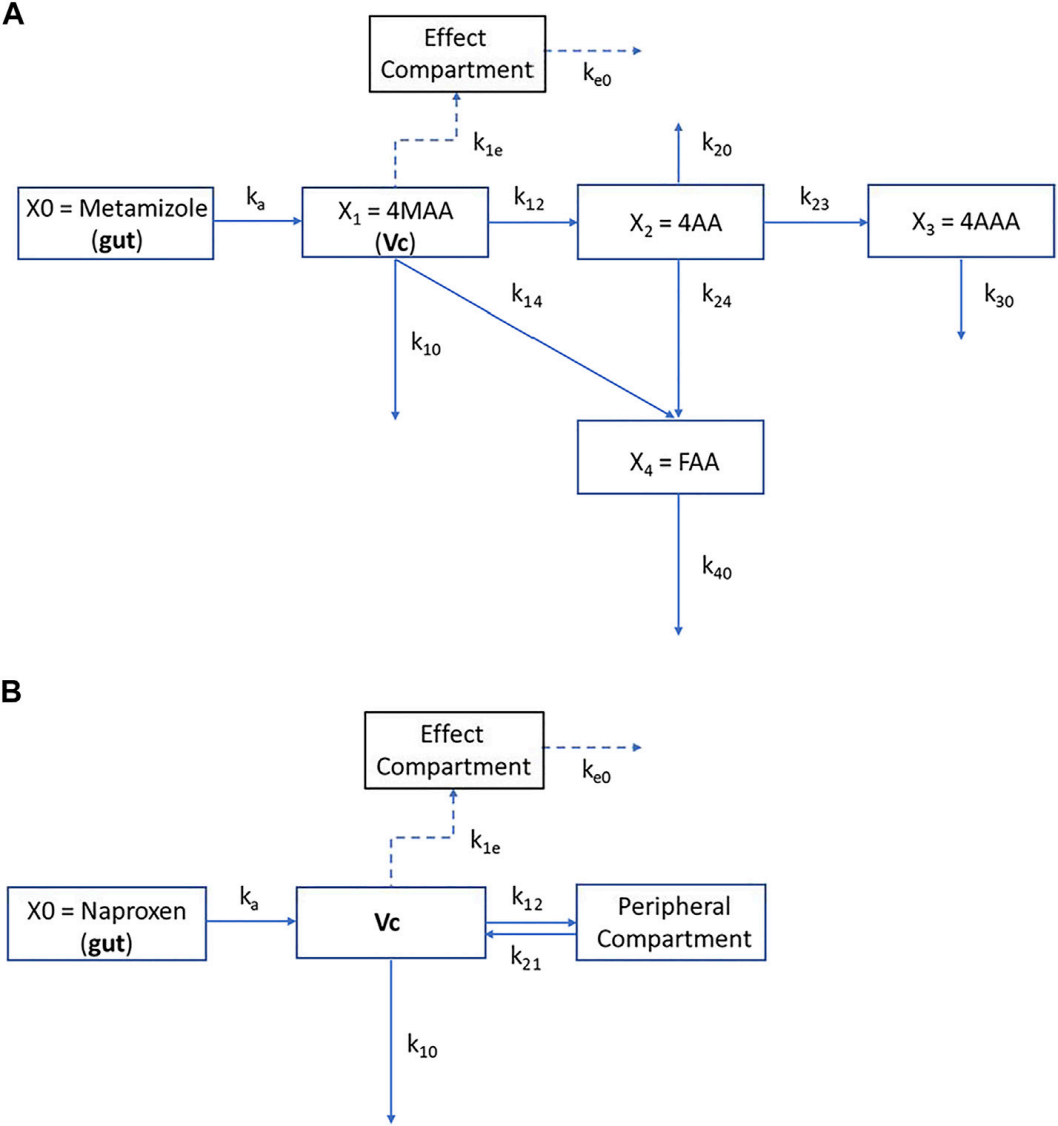
Compartmental structures of the PKPD models for metamizole metabolites **(A)** and naproxen **(B)**. Dosing of drugs was assumed to the gut compartment (X_0_). Concentration in this compartment was related to the central compartments V_C_ and X_1_ by a first-order rate constant (k_a_) that represented both dissolution and absorption from the gut into systemic circulation (naproxen) and in addition the formation of 4-MMA from metamizole, respectively. For metamizole the intercompartmental rate constants k_12_, k_21_, k_14_, k_23_, k_24_ represent the first-order interconversion rate constants from one metabolite species into another. The rate constants k_10,_ k_20_, k_30_, k_40_, are elimination rate constants from compartments X_1_ (4-MAA), X_2_ (4-AA), X_3_ (4-AAA), and X_4_ (4-FAA), respectively. The peripheral compartment for naproxen is denoted by 2. In both models the effect compartment was linked to the central compartment (X_1_ or V_c_) of the active metabolite 4-N-methylaminoantipyrine (4-MAA) or naproxen by the intercompartmental rate constant k_1e_ and k_eo_ was used as elimination rate constant from the effect compartment.

For the PKPD model, pharmacokinetics and pharmacodynamics of metamizole and naproxen were analyzed by compartmental methods. The respective differential equations were solved using the generalized least squares algorithm of the ADAPT 5 software (Biomedical Simulation Resource, University of Southern California, Los Angeles, CA, United States). The compartmental structures of the PKPD models used for metamizole and naproxen are shown in Figure 2.

The effect compartment was assumed to be linked to the X1 compartment (4-MAA) for metamizole and the central compartment (V_c_) for naproxen. The inhibitory effect on urinary 6-ketoPGF1a excretion was then related to the concentrations of the drugs in the effect compartment by the sigmoid Hill-equation:

$$Effect\;=\;E_{0}\;-\;\frac{E_{max\;}.\;C_{e}^{N}}{EC_{50}^{N}+\;C_{e}^{N}}\;\;\;$$

Where E_0_ denotes the initial effect, E_max_ the maximal effect, EC_50_ the concentration at half-maximal effect in the effect compartment and N the sigmoidicity coefficient. In both models, central compartment and effect compartment are linked with a first-order equilibration rate constant k_1e_ while k_e0_ denotes the elimination rate constant from the effect compartment.

### Statistical Analysis

No statistical hypothesis was set for this exploratory study. The sample size was chosen based on practical considerations. The random list was obtained using an open source random generator on Random.org. Statistical analyses were performed using GraphPad PRISM (Version 6, La Jolla, California, United States). For variables with normally distributed numerical values, arithmetic mean and standard deviations were calculated. For non-normally distributed variables, median and range were used. Urinary sodium excretion values were tested for between group differences using 2-way ANOVA with Holm-Šidak correction for multiple comparisons. Calculated inulin clearance values were parameterized by calculating areas under effect curve (AUEC) from time 0–5 h. AUECs between the metamizole and the naproxen group were statistically compared using a Mann-Whitney-U-test, whereas AUECs within a treatment group, but of different study days were tested using a Wilcoxon signed rank test. The course within a treatment group on a single study day was evaluated using a Friedman-test. Statistical differences were considered as significant when *p* ≤ 0.05.

## Results

Fifteen subjects completed the study. Demographic data and blood pressure values are shown in Supplementary Table S4. Eight subjects received metamizole, 7 subjects received naproxen. Two subjects allocated to naproxen dropped out before administration of the study drug. One subject withdrew consent, and the other subject developed an adverse reaction to inulin (nausea and vomiting, CTCAE grade 1). One of the two replacement subjects developed bronchospasm (CTCAE grade 3) after the start of the inulin infusion and was not replaced.

Median urinary sodium excretion was less than 60–80 mmol/day on day 3 and day 2 but exceeded the target range at the end of the treatment period (Table 1).

**TABLE 1:** Urinary sodium excretion in 24 h urine samples.

	Sodium excretion [mmol/day]	
	Metamizole (*n* = 8)	Naproxen (*n* = 7)
run-in period (day-4 to day-3)	52 (29–64)	43 (29–67)
start of treatment (day 1 to day 2)	53 (26–82)	52 (34–132)
end of treatment (day 7 to day 8)	85 (31–127)	104 (85–121)

Data are given as median (range).

**FIGURE 3:**
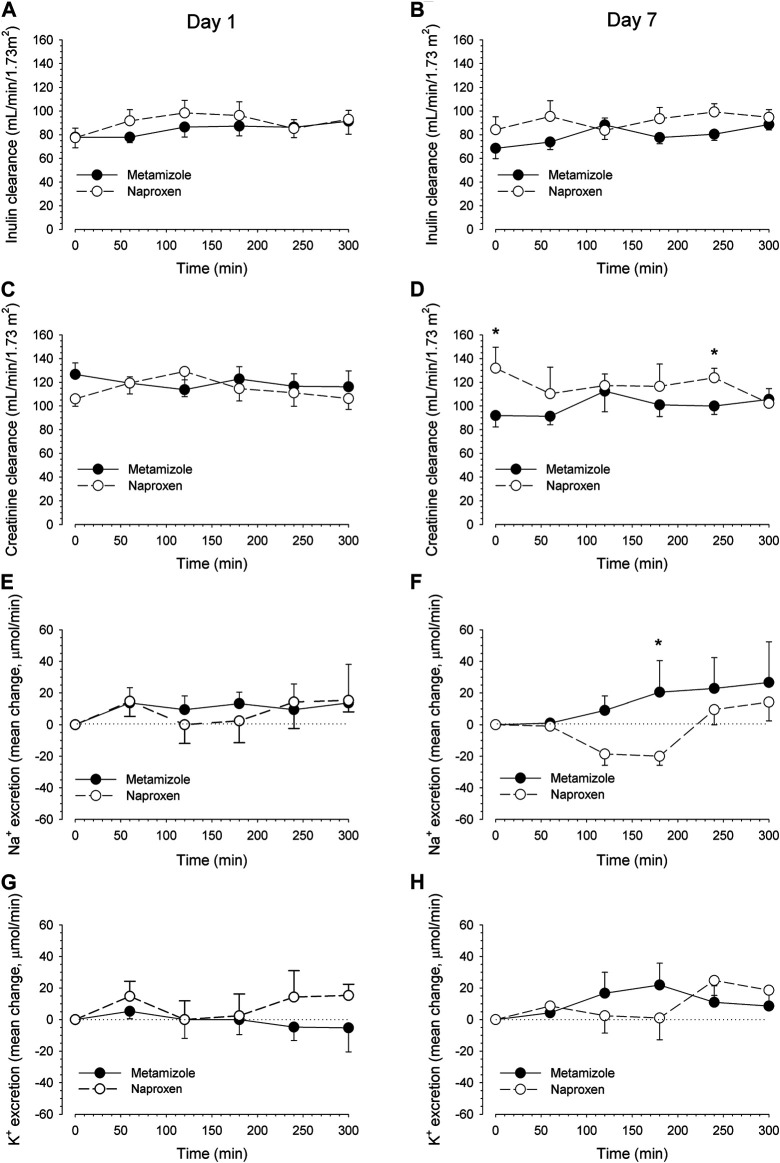
Effects on renal inulin and creatinine clearance and sodium and potassium excretion. The renal clearances of inulin (reflecting GFR) and creatinine after a single oral dose of 1 g metamizole and 500 mg naproxen (day 1, **left panels**) and after repetitive dosing at day 7 **(right panels)** are displayed in Figures 4A–D, respectively. Renal sodium excretion (Figures 4E,F) and potassium excretion (Figures 4G,H) as the difference to baseline excretion (time interval 08:00 to 09:00). The basal sodium excretion (mean ± SEM) was 40 ± 9 µmol/min and the basal potassium excretion 53 ± 12 µmol/min. Data are presented as mean values ±SEM. *p < 0.05 naproxen vs metamizole of the same time interval. There were no significant differences with baseline values within the different treatment groups.

Metamizole and naproxen had no significant effect on inulin or creatinine clearance both at day 1 and day 7 (Figures 3A–D). Creatinine excretion, for the completeness of bladder emptying during the urine collection periods, was not influenced by metamizole or naproxen and showed no significant differences among the collected urine fractions of the treatment groups (Supplementary Table S5).

Both drugs had only minimal and clinically not relevant effects on urinary sodium and potassium excretion (Figures 3E–H).

**FIGURE 4:**
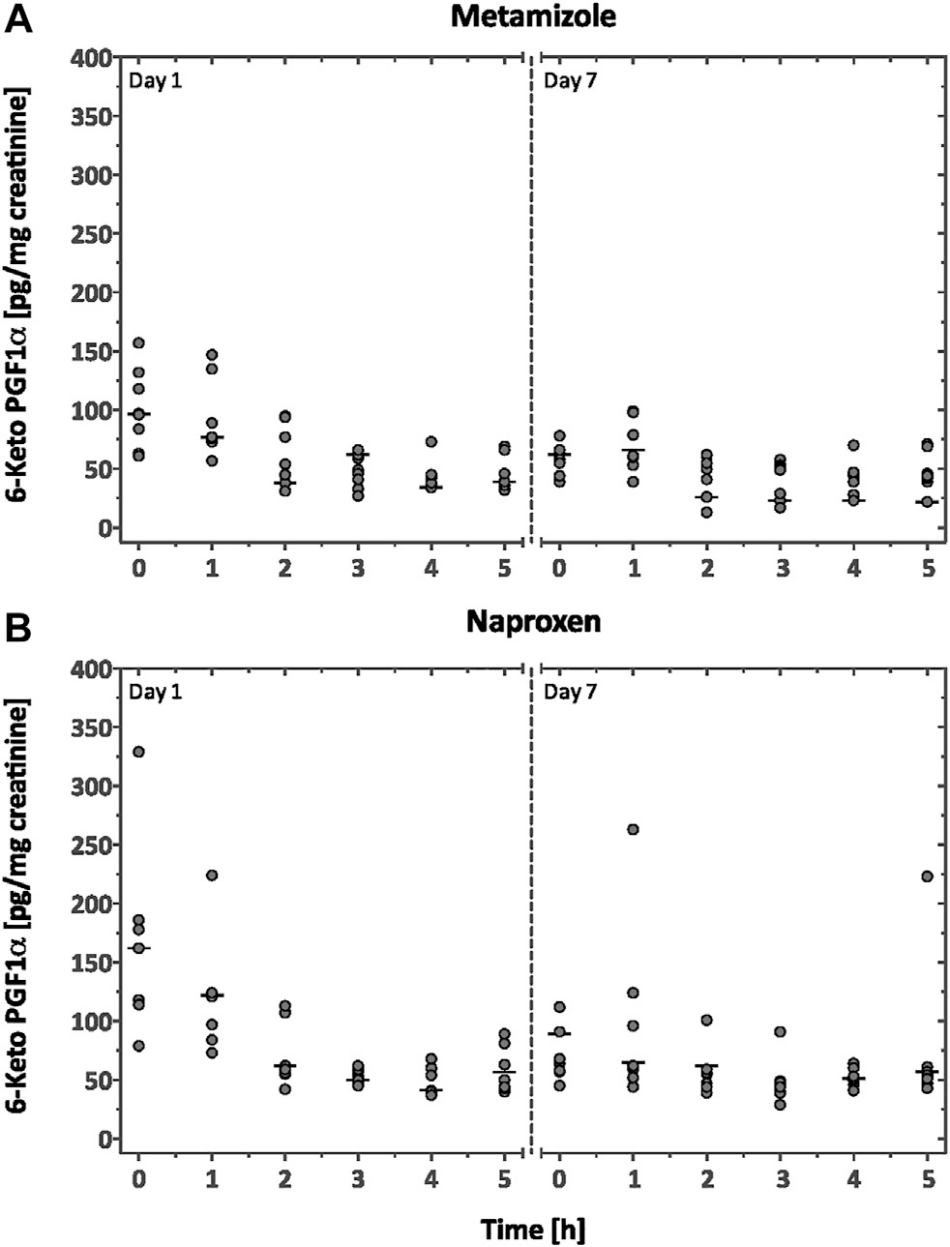
Urinary 6-keto-PGF1α excretion. Excretion of urinary 6-keto-PGF1α measured after a single oral dose of 1,000 mg metamizole **(A)** or 500 mg naproxen **(B)** on day 1 and after multiple daily dosing on day 7. Data are presented as individual data (filled circles) normalized to urinary creatinine content and median values (horizontal lines).

Naproxen and metamizole both decreased urinary 6-keto-PGF1α excretion. On day 1, before administration of the first dose, the urinary 6-keto-PGF1α concentrations in the baseline samples were higher than the lower limit of quantification of the analytical method for all subjects. Two hours after a single dose of metamizole or naproxen, the 6-keto-PGF1α concentrations started to decrease and remained low up to 5 h in most of the subjects (Figure 4A). At steady state (day 7), the trough value before administration of the morning dose was lower than the baseline value on day 1, indicating a long-lasting effect of both treatments on renal 6-keto-PGF1α excretion (Figure 4B). Concentrations of 6-keto-PGF1α decreased to levels below the limit of quantification (LLOQ, 8 pg/ml) after ingestion of metamizole in all subjects on day 1 and in 7 of 8 subjects on day 7. After naproxen this was the case for 5 of 7 subjects on day 1 and for all 7 subjects on day 7.

**FIGURE 5:**
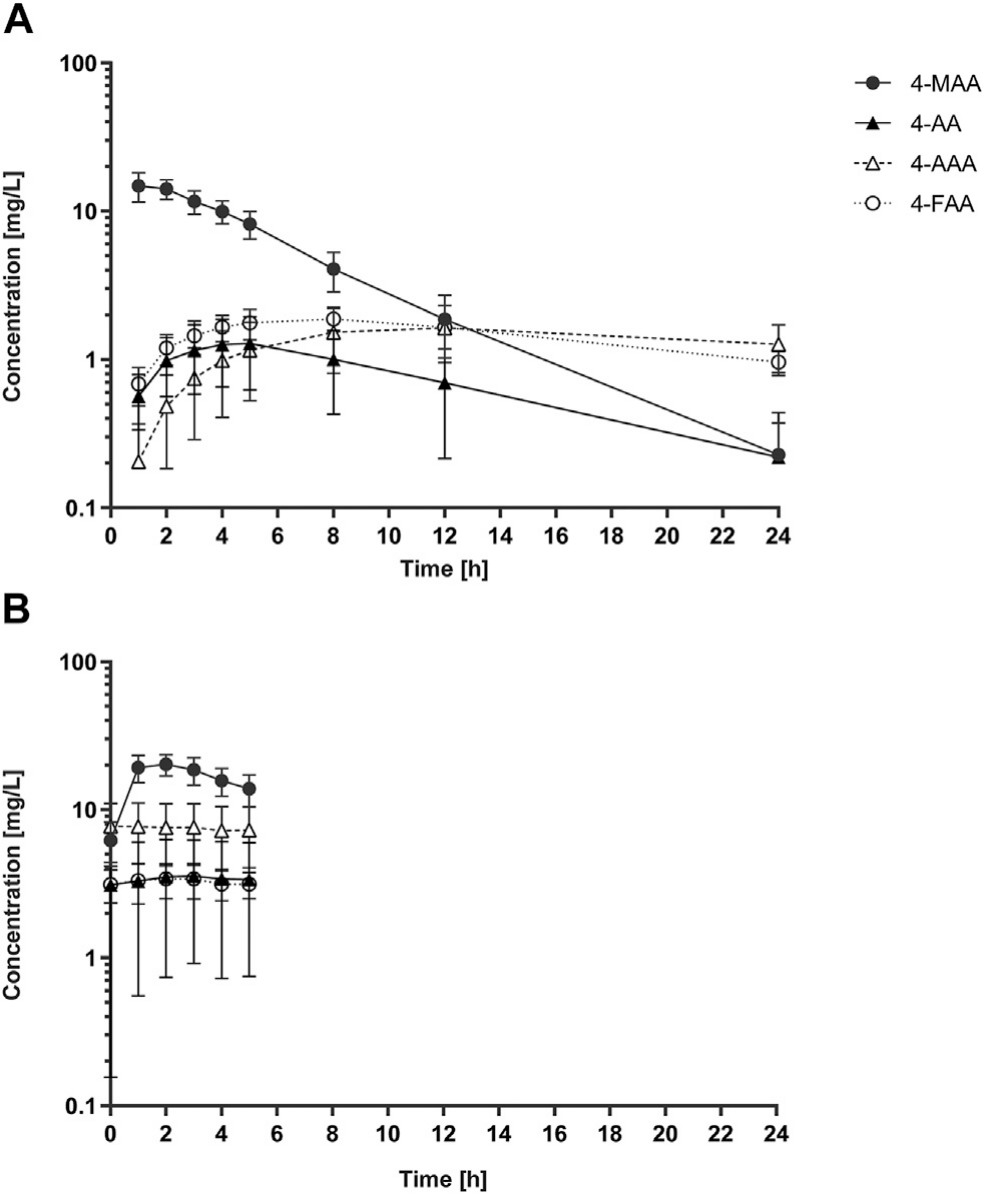
Pharmacokinetic profiles of metamizole metabolites. Plasma concentration vs time profiles of the four metamizole metabolites 4-MAA, 4-AA, 4-FAA and 4-AAA in healthy volunteers (*n* = 8) following a single oral dose of 1,000 mg metamizole **(A)** and after 7 days of continuous intake of 1,000 mg metamizole three times per day **(B)**. In panel **(B)** only data from the first dose interval (0–5 h) is shown. Data are presented as mean and SD.

The concentration-time profiles of the four main metamizole metabolites after single and multiple oral doses are shown in Figure 5 and the corresponding pharmacokinetic parameters are listed in Table 2. 4-MAA reached the highest plasma concentrations of all metabolites, both after single and multiple dose treatment. After single dose, the C_max_ values of the other metabolites 4-AA, 4-AAA and 4-FAA were 10–20 times lower compared with 4-MAA.

**TABLE 2:** Pharmacokinetic parameters after a single oral dose of 1,000 mg metamizole (*n* = 8) on day 1 and after multiple doses (1,000 mg TID) on day 7.

	4-MAA	4-AA	4-AAA	4-FAA
**Day 1**				
C_max_ [mg/L]	14.9 (11.9–18.5)	1.2 (0.6–2.5)	1.6 (0.8–2.5)	1.9 (1.4–2.3)
T_max_ [h]	1 (1–2)	5 (3–5)	12 (8–24)	8 (5–12)
AUC_0-24h_ [mg/L x h]	105 (67–128)	15 (7–31)	30 (15–471)	34 (27–40)
AUC_0-inf_ [mg/L x h]	107 (67–129)	16 (7–39)	-	65 (45–75)
AUC_0-5h_ [mg/L x h]	58 (48–70)	4.3 (2.3–8.8)	2.9 (1.0–5.2)	5.9 (4.2–7.6)
T_1/2_ [h]	3.7 (2.8–4.4)	5.8 (4.0–12.4)	-	16.0 (8.9–27)
**Day 7**				
C_max_ [mg/L]	21.2 (15.7–24.8)	3.0 (1.1–8.9)	8.3 (2.0–11.3)	3.5 (2.0–4.8)
C_max_ ratio MD/SD	1.35	2.22	4.85	1.77
AUC_0-5h_ [mg/L x h]	82.6 (55.1–106)	13.9 (4.8–42.4)	39.1 (9.2–53.9)	16.3 (8.7–22.0)
AUC_0-5h_ ratio MD/SD	1.42	3.24	13.35	2.77

Data are given as median and ranges. AUC_0-24h_, partial area under concentration-time curve from 0 to 24 h; AUC_0-5h_, partial area under concentration-time curve from 0 to 5 h (adapted to dosing interval); C_max_, maximum plasma concentration; MD, multiple dose; SD, single dose; T_1/2_, half-life; TID, three times per day; T_max_, time to maximum plasma concentration.

The concentration-time profiles of naproxen are shown in Supplementary Figure S2 and the corresponding pharmacokinetic data are listed in Supplementary Table S6.

**FIGURE 6:**
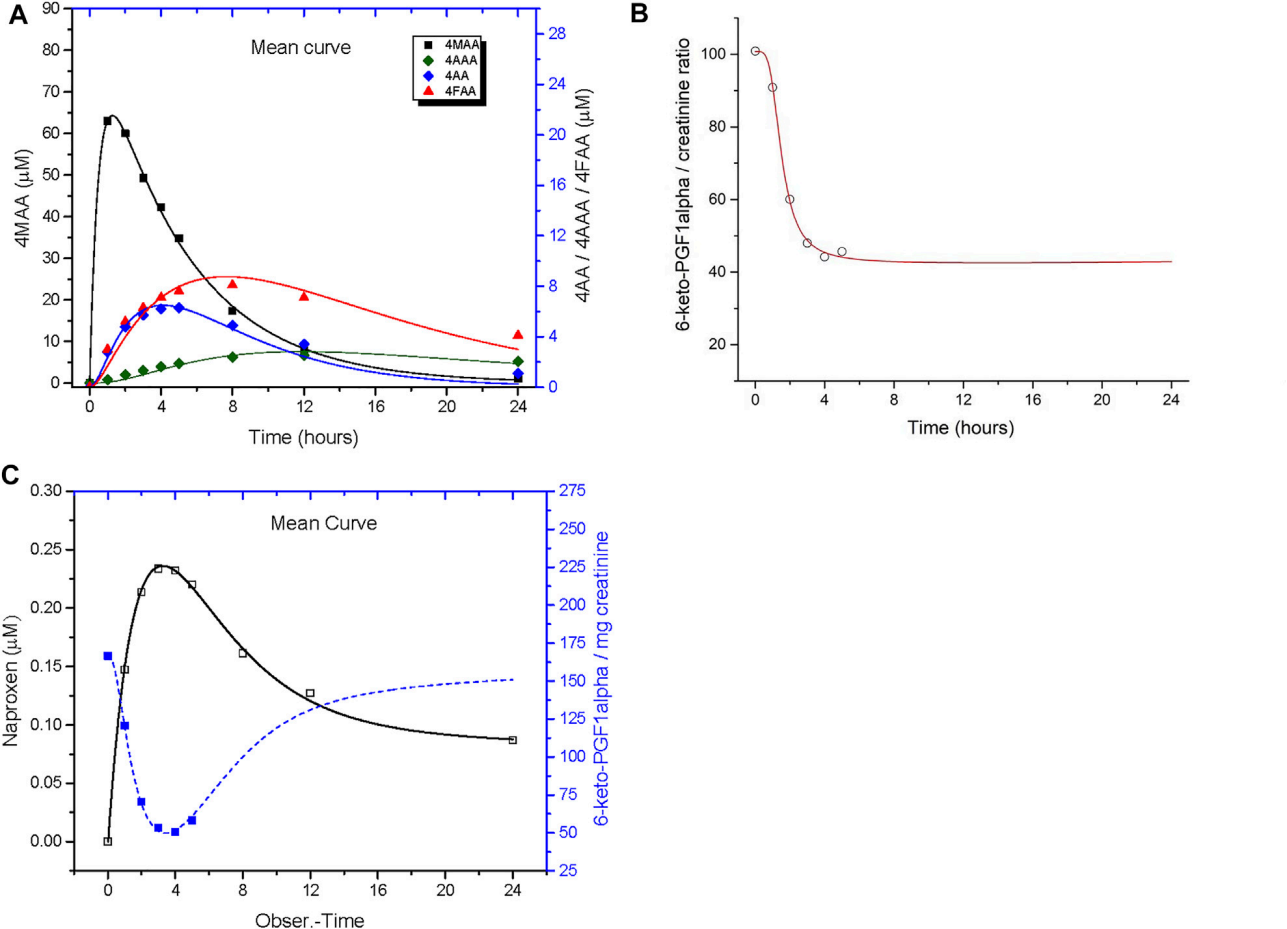
PKPD model output. Compartmental analysis of the pharmacokinetics and urinary 6-keto-PGF1α excretion after single dose treatment with 1,000 mg metamizole **(A,B)** and 500 mg naproxen **(C)**. The model is described in the methods section and the model parameters are given in Table 3. The individual fits are provided as supplementary material.

The results of the pharmacokinetic and pharmacodynamic models of metamizole and naproxen are given in Table 3 and Figure 6 and individual model fits are shown in Supplementary Materials. The plasma concentration-time curve of the primary metamizole metabolite 4-MAA and the effect of 4-MAA on the urinary excretion of 6-keto-PGF1α was well described by the model. The pharmacokinetics of the other three metamizole metabolites (4-AA, 4-FAA and 4-AAA) showed a lower agreement with the non-compartmental analysis. For naproxen, data from one subject led to unrealistic model output and had to be excluded, the averaged results of the remaining six subjects matched well with the non-compartmental analysis. Overall, the PKPD models indicated a significantly (*p* = 0.038) lower EC_50_ (0.034 vs 0.69 µM) and an approximately two-fold higher E_max_ (134 vs 61 pg 6-ketoPGF1α/mg creatinine) for naproxen compared with metamizole.

**TABLE 3:** Parameters of the pharmacokinetic and pharmacodynamic models for metamizole and naproxen. The model is described in the methods section. Values are presented as mean ± SEM.

Metamizole (*n* = 8)		Naproxen (*n* = 7)	
K_a_ (1/h)	2.528 ± 0.403	K_a_ (1/h)	0.538 ± 0.76
V1/F (L)	71.2 ± 3.0	Vc/F (L)	3.27 ± 1.12
k_12_ (1/h)	0.030 ± 0.010	K_10_ (1/h)	0.185 ± 0.096
k_14_ (1/h)	0.006 ± 0.002	k_12_ (1/h)	1.334 ± 0.677
k_10_ (1/h)	0.178 ± 0.025	k_21_ (1/h)	0.213 ± 0.073
V2/F (L)	35.6 ± 13.9	V2/F (L)	16.8 ± 5.58^a^
k_23_ (1/h)	0.552 ± 0.162	k_1e_ (1/h)	0.009 ± 0.005
k_24_ (1/h)	0.033 ± 0.025	k_eo_ (1/h)	2.275 ± 0.945
k_20_ (1/h)	0.008 ± 0.007		
V3/F (L)	55.1 ± 19.7		
k_30_ (1/h)	0.074 ± 0.008		
V4/F (L)	11.5 ± 5.1		
k_40_ (1/h)	0.567 ± 0.476		
k_e0_ (1/h)	0.012 ± 0.004		
T_1/2_ K01 (h)	0.36 ± 0.09	α (1/h)	1.708 ± 0.741
T_1/2_ 4-MAA (h)	3.43 ± 0.29	β (1/h)	0.028 ± 0.004^a^
T_1/2_ 4-AA (h)	2.39 ± 0.71	T_1/2_, K_a_ (h)	2.14 ± 0.63
T_1/2_ 4-AAA (h)	10.33 ± 1.25	T_1/2_, α (h)	1.00 ± 0.01
T_1/2_ 4-FAA (h)	6.95 ± 1.13	T_1/2_, β (h)	22.10 ± 1.32^a^
EC_50_ (µM)	0.69 ± 0.27	EC_50_ (µM)	0.034 ± 0.033
N	18.4 ± 9.0	N	7.2 ± 3.9
E_0_ ^b^	101.6 ± 11.9	E_0_	167.16 ± 33.68
E_max_ ^b^	61.3 ± 16.3	E_max_	133.56 ± 37.43

Ka, absorption rate constant; V1, Vc, volume of central compartments; V/F, apparent volume of distribution; α, distribution rate constant; β, terminal elimination rate constant, k_1e_, k_12_, k_14_, k_23_, k_24_, intercompartmental rate constants; k_10,_ k_20_, k_30_, k_40_, elimination rate constants; k_eo_, equilibration rate constant between central compartment and effect compartment; T_1/2_, half-life; EC_50_, concentration of half-maximal effect; E_max_, maximal effect; E_0_, initial effect; N, sigmoidicity coefficient of sigmoid Hill-equation.

a *n* = 6 (1 subject excluded due to unrealistic model output).

b Units for E_0_ and E_max_ are pg/mg creatinine (excretion of 6-ketoPGF1α normalized to creatinine content in urine).

## Discussion

In this study we investigated the effect of single and repeated doses of metamizole and naproxen on renal function in moderately salt depleted healthy human volunteers and we analysed the pharmacokinetics of the four main metamizole metabolites.

Under our experimental conditions, metamizole and naproxen had no clinically relevant effect on inulin or creatinine clearance.

Compared to the creatine clearance, the average inulin clearance in our study was approximately 20 ml/min lower. Creatinine clearances have been reported to exceed corresponding inulin clearances, possibly due to additional tubular creatinine secretion. In one study, the ratio of measured creatinine clearance to inulin clearance varied between 1.1 and 2.3, which is in agreement with our findings (van Acker et al., 1992). Incomplete emptying of the bladder or incomplete distribution of inulin after the 1 h equilibration period, causing variations in plasma concentration and renal excretion could be possible reasons for the low baseline values we observed. Since the formula to calculate inulin clearance relies on stable plasma levels over the entire observation period, insufficient equilibration is a possible cause for bias.

The lacking effect of naproxen on inulin clearance (and, therefore, GFR) was unexpected. In a previous clinical study with a comparable study design (except for a single dose furosemide pre-treatment), there was also no decrease in GFR after single dose of naproxen (Rossat et al., 1999). However, after repeated naproxen doses, a decrease in GFR of approximately 20% was observed (Rossat et al., 1999). The observation that our study subjects had a higher 24 h sodium excretion at the end compared to the start of the study, suggesting insufficient Na^+^ depletion, is a possible explanation for the lacking effect of naproxen on the GFR at day 7. Since our positive control naproxen did not significantly decrease inulin clearance, it is difficult to evaluate the effect of metamizole on GFR. At least under the conditions of our study, even repeated doses of metamizole did not decrease the inulin clearance. In 1995, Farker and colleagues investigated the influence of metamizole and diclofenac on glomerular filtration and renal perfusion in healthy male subjects by measuring the creatinine and inulin clearances and the clearance of para-aminohippurate (PAH) (Farker et al., 1995). In their study, neither metamizole, nor diclofenac at therapeutic doses over 3 days had a significant effect on GFR or renal plasma flow. Since diclofenac did not have an effect on these parameters also in other studies (Kinn et al., 1989; Fredman et al., 1999; Dilger et al., 2002; Farker et al., 2002; Whelton et al., 2006), it appears to be difficult to demonstrate the consequences of COX inhibition on renal function in healthy volunteers. Results from studies with naproxen were not consistent, with some studies showing a decrease in inulin clearance but not of the estimated creatinine clearance (Kamper et al., 1986; Eriksson et al., 1990; Huledal et al., 2005).

After a single dose, both metamizole and naproxen tended to increase renal sodium excretion, possibly due to the water load given to the study subjects (Anastasio et al., 2001; Wouda et al., 2019). After repeated dosing (and with the same oral water load as on day 1), sodium excretion decreased in the subjects treated with naproxen and increased in those treated with metamizole compared to baseline (Figures 3E,F). However, with the exception of the 3-h time-point, the differences were not statistically significant and can be considered as clinically not relevant. In a previous study (Rossat et al., 1999), decreased urinary sodium excretion had already been observed after a single dose of naproxen, possibly due to the more pronounced salt depletion (urinary sodium excretion 26–37 mmol/day vs 43–52 mmol/day) compared to our study.

The observed pharmacokinetic data of metamizole are in line with previously published values. The main metabolite 4-MAA showed a median half-life of 3.7 h, which is comparable to published values (Levy et al., 1995; Novalgin, 2015). The observed half-life for the other active metabolite 4-AA was comparable to values reported for slow N-acetylators (5.5 h, vs 3.8 h for rapid acetylators (Novalgin, 2015)). The C_max_ of 4-MAA observed in the current study was in the higher range (14.9 mg/L in current study compared to 9.7–17.3 mg/L) and the AUC_0-inf_ was slightly above previously reported values (107 mg/L x h in the current study compared to 64.5–95.1 mg/L x h (Levy et al., 1995)). The ratios of C_max_ and AUC_0-5h_ after repeated dosing compared with values after single doses indicated 1.4 and 3.2-fold accumulation of the active metabolites 4-MAA and 4-AA, respectively. This is compatible with the half-lives of the two metabolites in relation to the dosing interval of 8 h 4-MAA reached approximately 7 to 10-fold higher values for C_max_ and AUC compared to 4-AA and it is therefore primarily responsible for the analgesic effect of metamizole both after single and repeated dosing. Regarding toxicity, whose mechanisms are currently not well established, the other metabolites may also contribute.

After single and multiple dose treatment, metamizole and naproxen both reduced renal 6-keto-PGF1α excretion, indicating COX-inhibition by both drugs (Figure 4). The reduction of the urinary 6-keto-PGF1α excretion started 2 h after drug administration, which corresponds well with the T_max_ of naproxen and the active metamizole metabolite 4-MAA, but not with the time course of the other metamizole metabolites. The effect lasted for the whole observation period except for a few subjects, who showed a moderate increase of 6-keto-PGF1α excretion towards the end of the observation period. Before ingestion of the morning dose on day 7, 6-keto-PGF1α excretion was lower compared to the baseline on day 1 in both groups, indicating persistent COX-inhibition 12 h after intake of the previous drug dose. As concentrations below the LLOQ of the bioanalytical method were set to the LLOQ value, the observed effect size is a conservative estimate of the true effect. For most subjects, the urine creatinine concentrations in the 2–5 h fractions were comparable to the baseline fraction, indicating that the observed decrease of 6-keto-PGF1α concentrations after metamizole or naproxen was not caused by urine dilution.

According to the EC_50_ values obtained from the PKPD models, 4-MAA was a less potent (i.e., higher EC_50_ value) inhibitor of prostacyclin formation compared with naproxen, which had an approx. 20-fold lower EC_50_. Although plasma concentrations of 4-MAA were much higher after therapeutic doses of metamizole than those of naproxen, they both were well above the corresponding EC_50_ values obtained by the PKPD models, which is compatible with the comparable effect sizes observed for the two drugs. As the concentrations of 6-keto-PGF1α decreased to levels below the quantification limit of the analytical method in most of the subjects within a few hours after dosing, the lower plateau (or maximal effect) could not be quantified precisely, but given the low LLOQ of the method, the possible error is small. The differences in observed maximal effect (Emax = efficacy) thus primarily come from differences in the baseline concentrations of 6-keto-PGF1α in the two small groups and, overall, the two drugs appear to have a roughly comparable effect size.

Both PKPD models assume that the effect in the effect compartment is linked to the concentration of the active substance in the central compartment (Vc). In the case of naproxen, the active substance is naproxen itself; in the case of metamizole, it is the metabolite 4MAA. As PK data of the other metamizole metabolites (4AA, 4AAA, 4FAA) was available, the PKPD model used for naproxen was expanded to includes these metabolites. However, the changes only concern the peripheral compartment and the other metamizole metabolites are not needed to explain the effect of metamizole.

Despite the reduction of 6-keto-PGF1α excretion in both groups, we did not observe a reduction in the glomerular filtration rate. The most likely explanation is that our study subjects were not sufficiently sodium depleted to make glomerular perfusion dependent on renal synthesis of vasodilatory prostaglandins such as prostacyclin. A decrease in renal prostaglandin excretion after metamizole intake has been described in previous studies with healthy volunteers (Rosenkranz et al., 1992) and in patients with liver cirrhosis with or without ascites (Zapater et al., 2015). In these patients, metamizole did not reduce renal function when used for a short time period of 72 h. Since patients with liver cirrhosis are at increased risk for renal failure after inhibition of prostaglandin synthesis (Imani et al., 2014; Elia et al., 2015), these results support better renal tolerability of metamizole compared to conventional NSAIDs in patients with reduced intravascular volume. Production of vasodilatory prostacyclin is an important counter-regulatory mechanism to maintain renal perfusion in states of intravascular volume contraction. As synthesis of prostacyclin seems to be similarly affected by metamizole as by naproxen, other mechanisms may to be responsible for the better renal tolerability of metamizole compared to classic NSAIDs observed in clinical practice. One possible mechanism could be COX-independent direct smooth muscle relaxant effects of metamizole which have previously been described in various animal models (Ergün et al., 1999; Ergün et al., 2000; Valenzuela et al., 2005; Gulmez et al., 2006; Gülmez et al., 2008; Silav et al., 2017).

A limitation of our study is the lack of the positive control naproxen to reduce inulin clearance, most likely due to insufficient sodium depletion of our study subjects at the end of the repeated dosing period. Further limitations are the lack of a placebo group which would have helped to differentiate the effect of the water load on inulin clearance and renal sodium excretion and the uncertainties in the model estimates of individual pharmacodynamic effects. In addition, we did not assess the renal blood flow, which might have shown an effect of naproxen.

In conclusion, metamizole had a comparable effect on renal excretion of the prostacyclin metabolite 6-keto-PGF1α and thus seems to inhibit renal prostacyclin synthesis to a similar degree as the non-specific NSAID naproxen. Despite reducing renal 6-keto-PGF1α excretion, none of the drugs had a significant effect on glomerular filtration rate or sodium and potassium excretion in healthy, moderately sodium-depleted subjects. Possible mechanisms for the better renal tolerability of metamizole compared to conventional NSAIDs observed in clinical practice such as direct vasodilatory effects counteracting the reduced levels of vasodilating prostacyclin remain to be investigated.

## Data Availability

The raw data supporting the conclusions of this article will be made available by the authors, without undue reservation.

## Abbreviations

4-AA, 4-aminoantipyrine; 4-AAA 4-N-acetylaminoantipyrine; 4-FAA, 4-N-formylaminoantipyrine; 4-MAA, 4-N-methylaminoantipyrine; AUC, area under the concentration–time curve; AUC_0-5h_, partial area under the concentration-time curve from 0 to 5 h; AUC_0-24h_, partial area under the concentration-time curve from 0 to 24 h; AUEC, area under effect curve; COX, cyclooxygenase; CTCAE, common terminology criteria for adverse events; E_0_, initial effect; EC_50_, concentration of half-maximal effect; E_max_, maximal effect; GFR, glomerular filtration rate; IC_50_, half maximal inhibitory concentration; NSAID(s), nonsteroidal anti-inflammatory drug(s).
