# Supplementary material for: Comparative Effects of Metamizole (Dipyrone) and Naproxen on Renal Function and Prostacyclin Synthesis in Salt-Depleted Healthy Subjects - A Randomized Controlled Parallel Group Study
Source: Front Pharmacol. 2021 Sep 7;12:620635. doi: 10.3389/fphar.2021.620635 (PMC8453264; doi:10.3389/fphar.2021.620635)
Supplement: Supplementary file 1 [file DataSheet1.pdf]

Comparative renal effects of metamizole (dipyrone) and naproxen in salt-depleted healthy subjects, a randomized controlled parallel group study

Supplementary Material

Supplementary Table 1 Inclusion / Exclusion criteria

INCLUSION CRITERIA

- Healthy male subjects aged between 18 and 45 years (inclusive) at screening
- BMI between 18 and 28 kg/m2 (inclusive) and body weight at least 50 kg at screening.
- SBP: 100-140 mmHg, DBP: 60-90 mmHg and HR: 45-90 bpm (inclusive), measured on the leading arm*, in the supine position at screening.
- No clinically significant findings on the physical examination at screening.
- 12-lead ECG without clinically relevant abnormalities at screening.
- Signed informed consent prior to any study-mandated procedure.
- Hematology and clinical chemistry results not deviating from the normal range to a clinically relevant extent at screening.
- Ability to communicate well with the investigator and to understand and comply with the requirements of the study.

*leading arm right = writing with right hand

EXCLUSION CRITERIA

- Smoking > 5 cigarettes per day.
- History or clinical evidence of alcoholism or drug abuse within the 3-year period prior to screening.
- Loss of ≥ 250 ml of blood within 3 months prior to screening.
- Treatment with an investigational drug within 30 days prior to screening.
- Previous treatment with any prescribed or OTC medication (including herbal medicines such as St John’s Wort) within 2 weeks prior to the intended start of the study.
- Legal incapacity or limited legal capacity at screening.
- Positive results from urine drug screen at screening.
- History or clinical evidence of any disease (e.g. GIT-disease: Morbus Crohn, Colitis Ulcerosa, anamnestic gastrointestinal bleeding) and/or existence of any surgical or medical condition, which might interfere with the absorption, distribution, metabolism or excretion of the study drugs, or which might increase the risk for toxicity.
- Known hypersensitivity to Aspirin or other NSAIDs or any excipients of the drug formulations.
- Known food allergy, especially intolerance to root vegetables (e.g. celeriac)
- Any circumstances or conditions, which, in the opinion of the investigator, may affect full participation in the study or compliance with the protocol.

Supplementary Table 2 Visit and assessment schedule

Study Period	SCR	Na-DEPL	TREATMENT PERIOD																						
**Study Day**	-21 - 8	-7-1	1*										2	3-6	7										8 (EOS)
Time after drug administration (h)			-2	-1	0	1	2	3	4	5	8	12			-2	-1	0	1	2	3	4	5	8	12	
Approximate clocktime (hh:min)			07:00	08:00	09:00	10:00	11:00	12:00	13:00	14:00	17:00	21:00	09:00	08:00	07:00	08:00	09:00	10:00	11:00	12:00	13:00	14:00	17:00	21:00	09:00
Informed consent, medical history, body weight and height	X																								
Physical examination +ECG	X																								X
Previous/concomitant medication & adverse events	X			X									X			X									X
Hematology & Clinical chemistry	X		X[13]												X[13]										X
Urine drug screen	X[1]																								
BP/HR	X			X												X									X
Inulin priming Infusion (125 mg/min over 20 min)			X												X										
Inulin constant infusion (28mg/min over 400			X[6]	-	-	-	-	-	-	End					X[6]	-	-	-	-	-	-	End			
Blood sampling Inulin-Cl			X[2]	X	X	X	X	X	X	X					X[2]	X	X	X	X	X	X	X			
Blood sampling for PK					X	X	X	X	X	X	X	X	X				X	X	X	X	X	X	X	X	X
Urinary PGE2 & 6-keto-PGF1alpha, Na^+ & K^+,				X[9]	X	X	X	X	X							X[9]	X	X	X	X	X				
Urine collection 24 h [Na^+, K^+]		X[8]		X[4]									X[5]			X[4]									X[5]
Drug administration					X								X[12]	X[12]			X					X[10]		X	
Overnight fast			X												X										
Water load 700mL during 30 min			X												X										
Oral Hydration 300mL/h			X[11]	X	X	X	X	X	X						X	X	X	X	X	X	X				
Provided meal at the University Hospital		X[3]								X[3]			X[3]	X[3]								X[3]			

Supplementary Table 3 Mass transitions and compound specific settings

ID	Q1 Mass (Da)	Q3 Mass (Da)	Time (msec)	DP (V)	CE (V)	CXP (V)	EP (V)
**4-AA**	204.2	56.2	30	71	39	8	10
**4-AA-d3**	207.2	59.1	30	46	39	10	10
**4-AAA**	246.2	83.1	30	46	43	14	10
**4-AAA-d3**	249.2	84.1	30	61	43	6	10
**4-FAA**	232.2	204.1	30	46	19	12	10
**4-MAA**	218.2	56.1	30	31	16	10	10
**4-MAA-d3**	221.2	56.1	30	46	37	8	10
**Naproxen**	229.1	168.8	50	-40	-36	-15	-10
**Naproxen-d3**	232.1	170.8	50	-45	-40	-9	-10
**6-Keto PGF1α**	564.1	306.1	100	-155	-36	-21	-10
**6-Keto PGF1α-d4**	568.1	310.1	100	-155	-36	-21	-10
**2,3-Dinor 6-Keto PGF1α-d9**	545.2	259.0	100	-210	-28	-17	-10

Supplementary Table 4 Demographic and clinical data

Age (years)	25 (18 – 29)	
Body weight (kg)	72.0 (57.4 – 97.1)	
BMI (kg/m^2)	22.2 (18.6 – 27.8)	
	Day 1*	Day 7*
Systolic blood pressure (mmHg)	125 (110-146)	125 (104 – 138)
Diastolic blood pressure (mmHg)	76 (66 – 93)	75 (60 – 87)
Serum creatinine concentration (µmol/L)	75 (58 – 95)	81 (62 – 100)

Data are given as median (range). N=15 study subjects.

* before administration of inulin

Supplementary Table 5 Creatinine excretion (μmol/h) by subjects treated with metamizole (n=8) or naproxen (n=7). Values represent means ± SEM.

	Metamizole		Naproxen	
Collection period	Day 1	Day 7	Day 1	Day 7
08:00 -09:00	686±64	545±63	496±39	628±58
09:00 - 10:00	649±54	544±51	551±31	536±117
10:00 - 11:00	622±68	652±82	591±84	578±65
11:00 -12:00	671±80	601±71	536±50	566±100
12:00 - 13:00	625±63	593±48	510±42	601±48
13:00 - 14:00	632±91	631±39	487±29	511±72

Supplementary Table 6 Pharmacokinetic parameters after a single oral dose of 500 mg naproxen (n=7) on day 1 and after multiple doses (500 mg BID) on day 7.

	**Naproxen**
**Day 1**	
T1/2 [h]	18.4 (12.8-21.8)
Tmax [h]	2(2-5)
Cmax [mg/L]	63.2 (51.7-76.0)
$AUC_{0\text{-inf\_obs}}$ [mg/L*h]	1293 (889-1528)
$AUC_{0\text{-12h}}$ [mg/L*h]	497 (398-560)
**Day 7**	
Tmax [h]	2 (1-3)
Cmax [mg/L]	85.7 (67-101)
Cmax ratio MD/SD	1.37
$AUC_{0\text{-12h}}$ [mg/L*h]	754 (572-817)
$AUC_{0\text{-12h}}$ ratio MD/SD	1.52

Data are given as median and range. $AUC_{0\text{-inf\_obs}}$ area under the concentration-time curve from zero to infinity extrapolated based on the last observed concentration; $AUC_{0\text{-12h}}$, partial area under concentration-time curve from 0 to 12 hours; C_{max}, maximum plasma concentration; MD, multiple dose; SD, single dose; $T_{1/2}$, half-life; BID, twice a day; T_{max}, time to maximum plasma concentration.

Supplementary Figure 1 Study Design

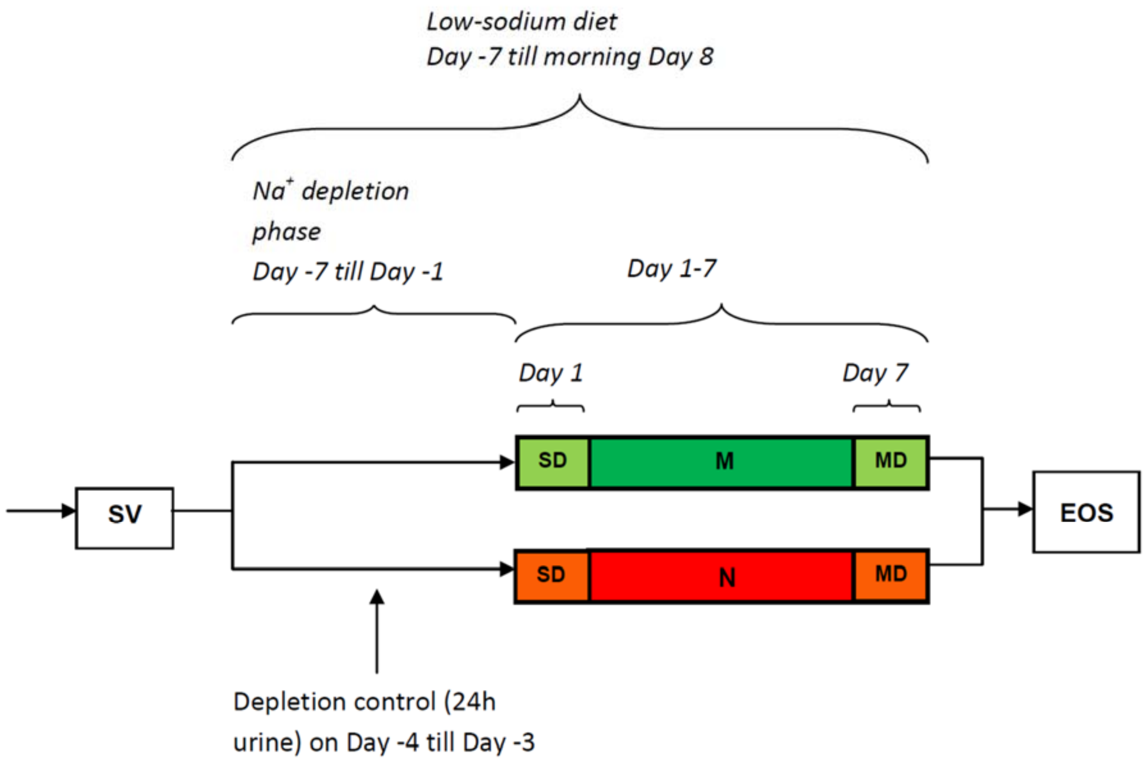

EOS	end of study visit
MD	multiple dose
M	metamizole
N	naproxen
SD	sinlge dose
SV	screening visit

Supplementary Figure 2 Mean plasma concentration of naproxen vs. time in healthy volunteers (n= 7) following a single oral dose of 500 mg (A) and after seven days of continuous intake of 500 mg twice a day (B). Due to an additional naproxen dose 12 hours after the morning dose, the PK profile is only shown for the first 12 hours.

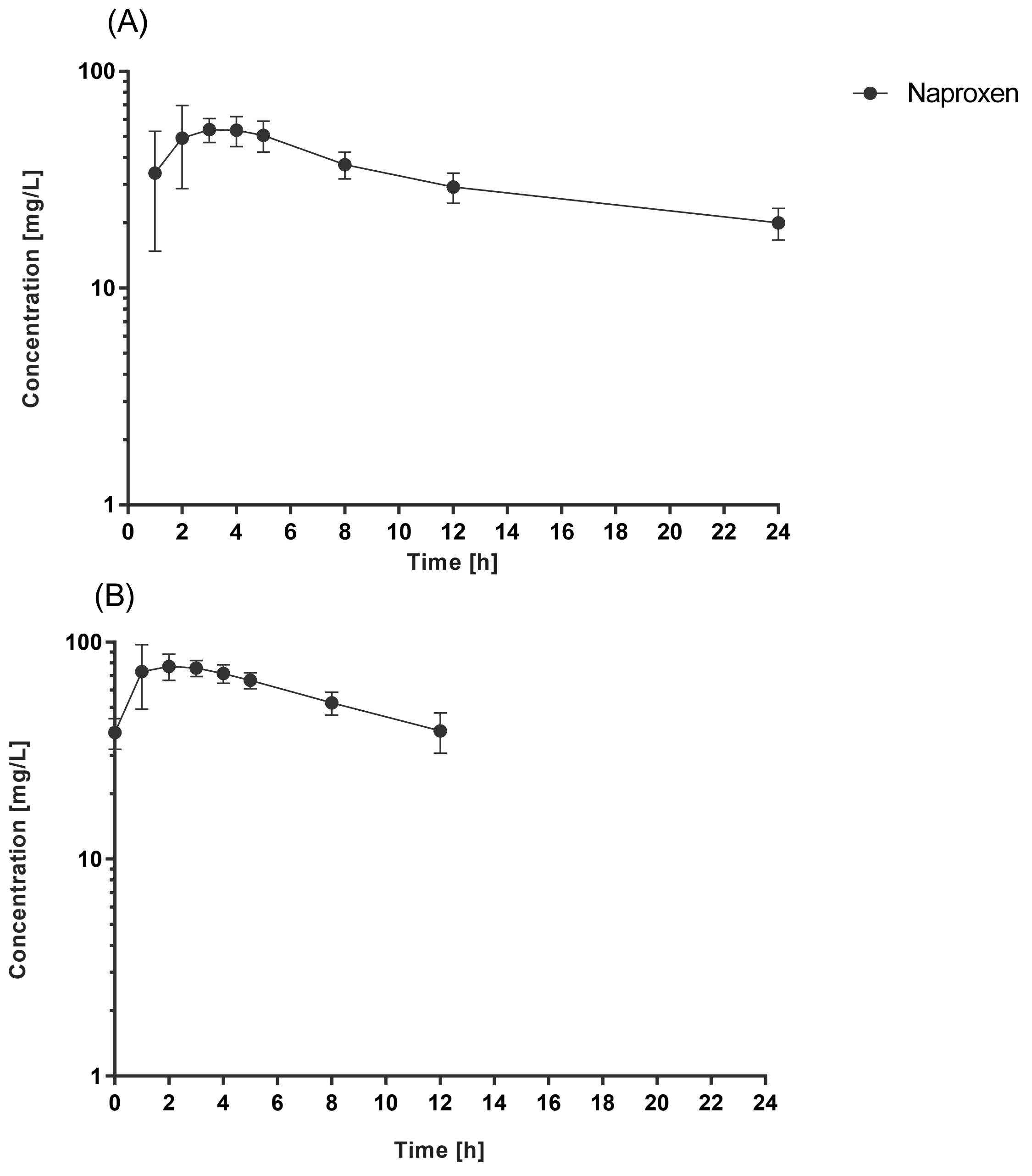

Individual pharmacokinetic and pharmacodynamic profiles Metamizole

Subject 1

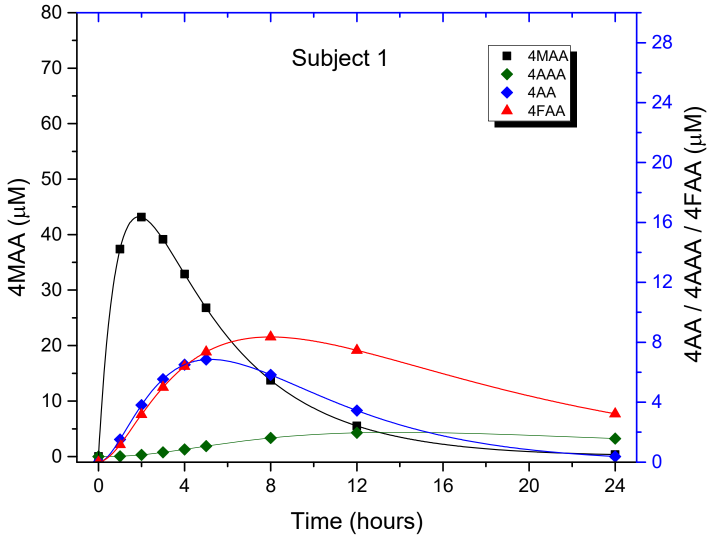

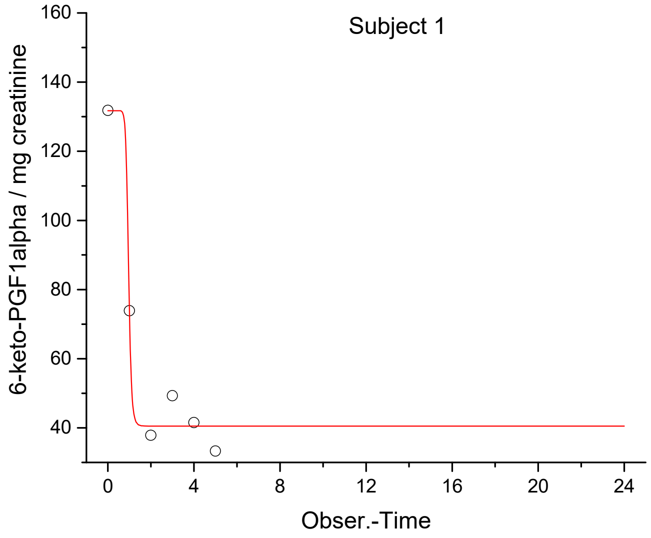

Units for pharmacodynamic effect are uinary exretion of 6-keto-PGF1α in pg/mg creatinine

Subject 4

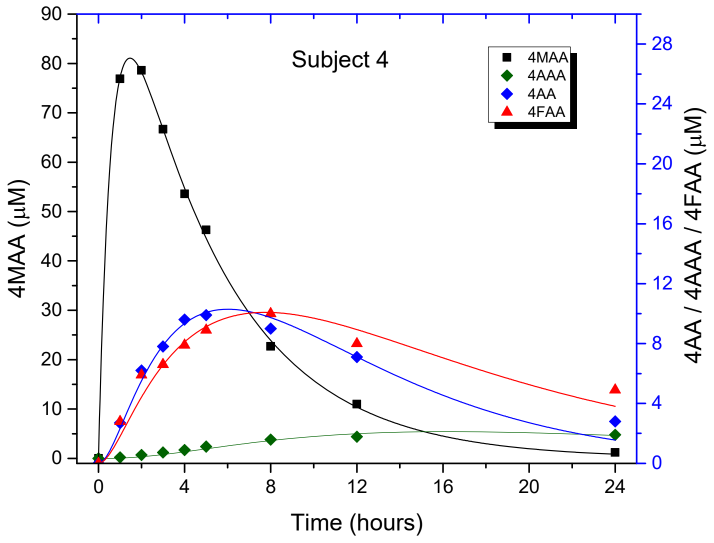

Subject 4
4MAA
4AAA
4AA
4FAA
4MAA (μM)
4AA / 4AAA / 4FAA (μM)
Time (hours)
0
4
8
12
16
20
24
0
10
20
30
40
50
60
70
80
90
0
4
8
12
16
20
24
28

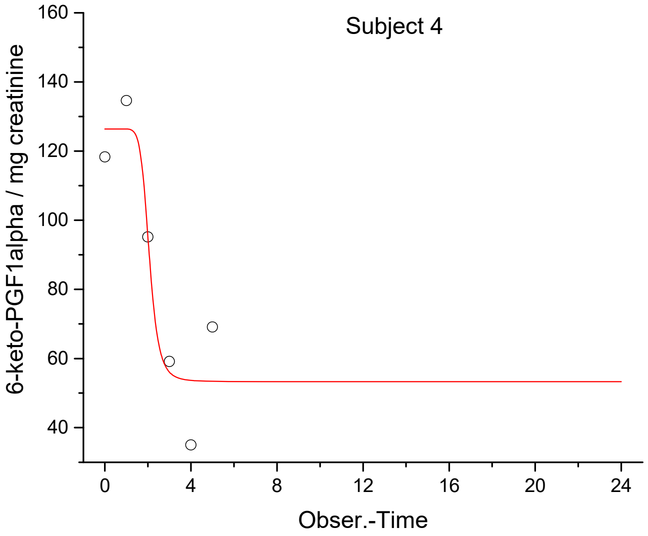

Subject 4
6-keto-PGF1alpha / mg creatinine
Obser.-Time
40
60
80
100
120
140
160
0
4
8
12
16
20
24

Subject 5

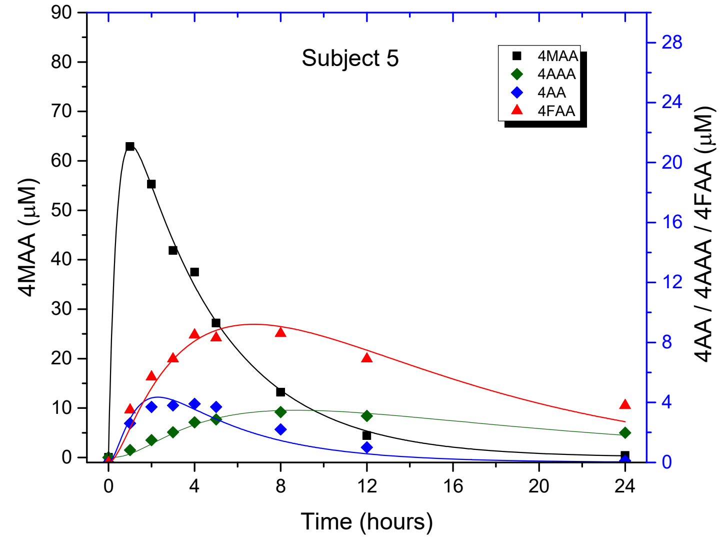
Subject 5
4MAA
4AAA
4AA
4FAA
4MAA (µM)
4AA / 4AAA / 4FAA (µM)
Time (hours)
0
4
8
12
16
20
24
0
10
20
30
40
50
60
70
80
90
0
4
8
12
16
20
24
28

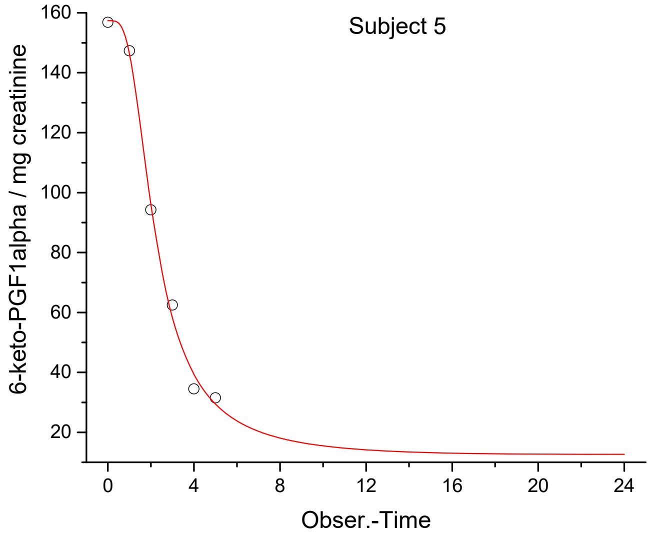
Subject 5
6-keto-PGF1alpha / mg creatinine
Obser.-Time
0
4
8
12
16
20
24
20
40
60
80
100
120
140
160

Subject 9

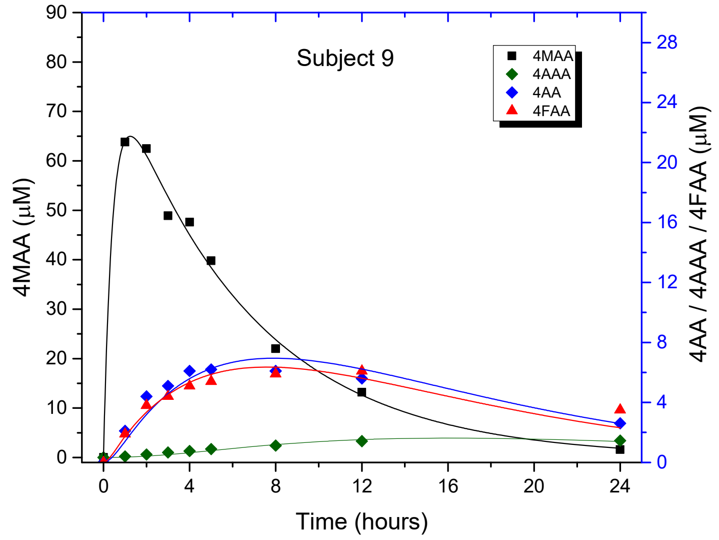

Subject 9
4MAA
4AAA
4AA
4FAA
4MAA (µM)
4AA / 4AAA / 4FAA (µM)
Time (hours)
0
4
8
12
16
20
24
0
10
20
30
40
50
60
70
80
90
0
4
8
12
16
20
24
28

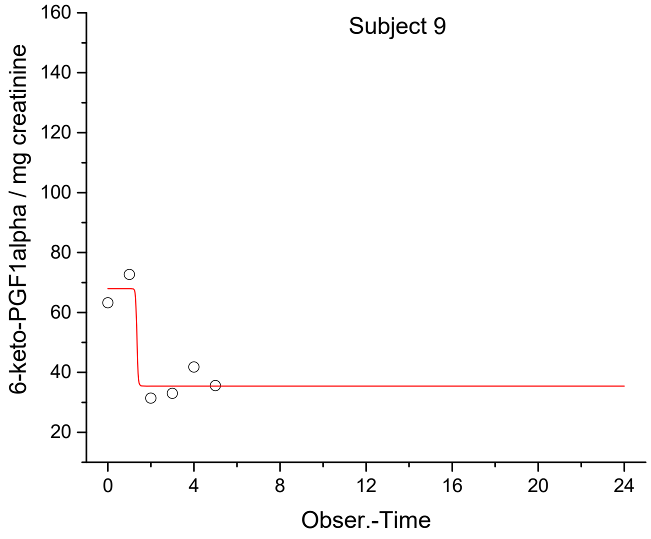

Subject 9
6-keto-PGF1alpha / mg creatinine
Obser.-Time
0
4
8
12
16
20
24
20
40
60
80
100
120
140
160

Subject 10

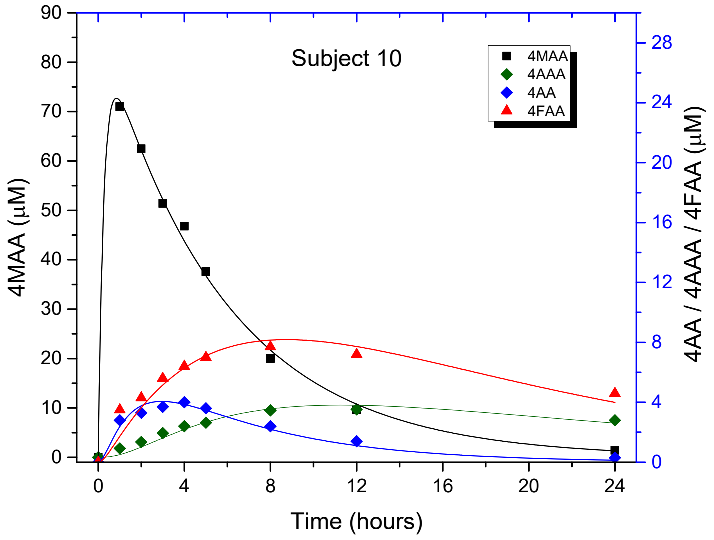
Subject 10
4MAA
4AAA
4AA
4FAA
4MAA (µM)
4AA / 4AAA / 4FAA (µM)
Time (hours)
0
4
8
12
16
20
24
90
80
70
60
50
40
30
20
10
0
28
24
20
16
12
8
4
0

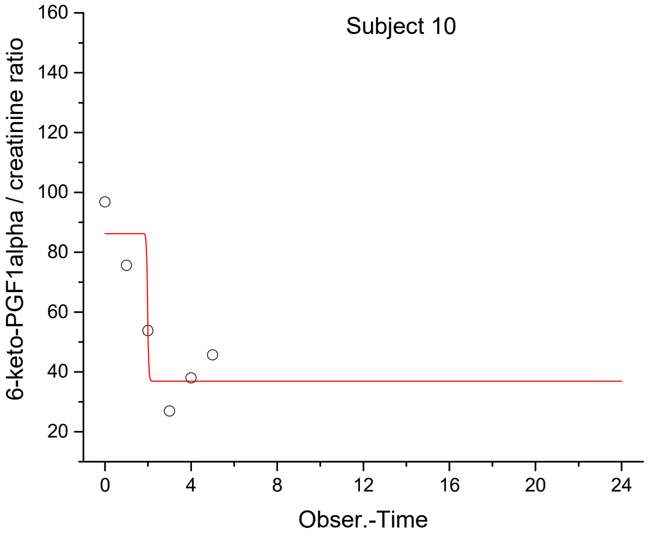
Subject 10
6-keto-PGF1alpha / creatinine ratio
Obser.-Time
160
140
120
100
80
60
40
20
0
4
8
12
16
20
24

Subject 11

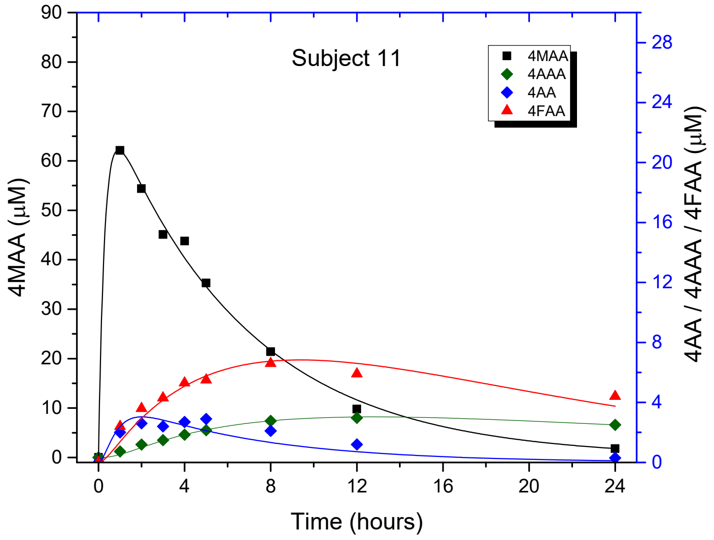
Subject 11
4MAA
4AAA
4AA
4FAA
4MAA (µM)
4AA / 4AAA / 4FAA (µM)
Time (hours)
0
10
20
30
40
50
60
70
80
90
0
4
8
12
16
20
24
28
0
4
8
12
16
20
24

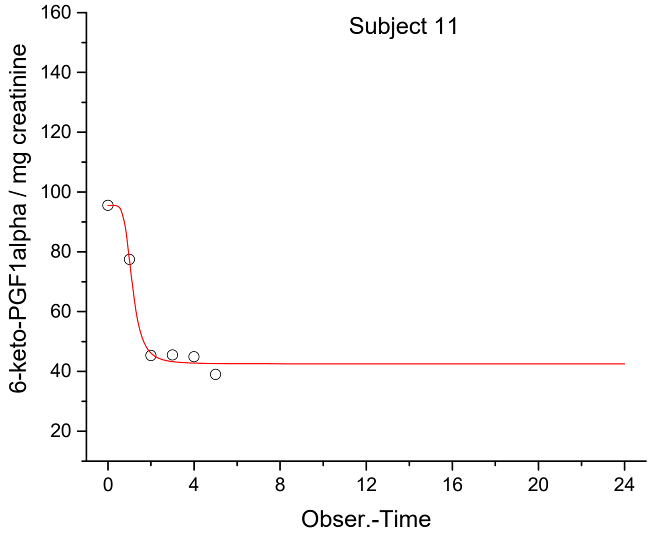
Subject 11
6-keto-PGF1alpha / mg creatinine
Obser.-Time
20
40
60
80
100
120
140
160
0
4
8
12
16
20
24

Subject 13

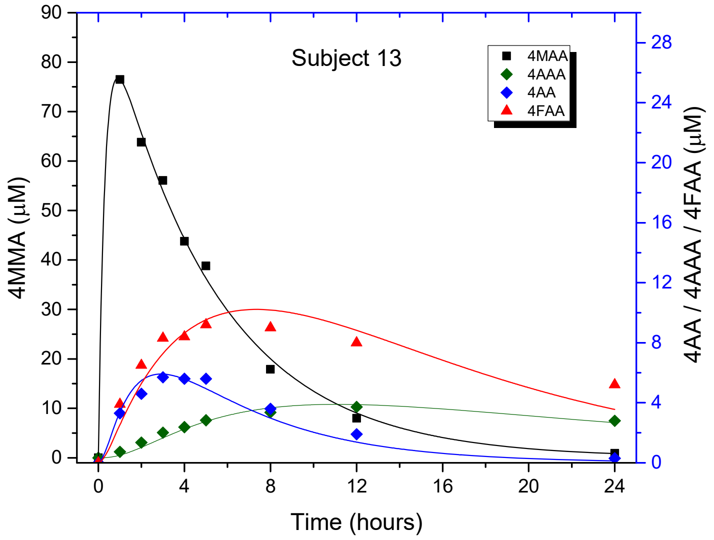

Subject 13
4MAA
4AAA
4AA
4FAA
4MMA (µM)
4AA / 4AAA / 4FAA (µM)
Time (hours)
90
80
70
60
50
40
30
20
10
0
28
24
20
16
12
8
4
0
0
4
8
12
16
20
24

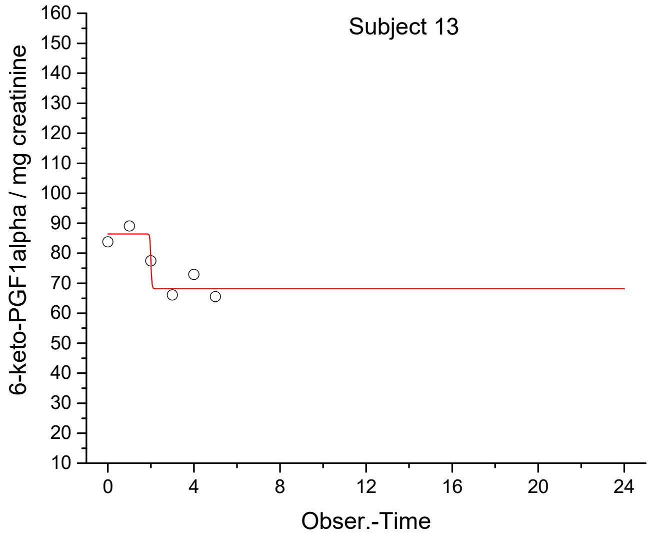

Subject 13
6-keto-PGF1alpha / mg creatinine
Obser.-Time
160
150
140
130
120
110
100
90
80
70
60
50
40
30
20
10
0
4
8
12
16
20
24

Subject 15

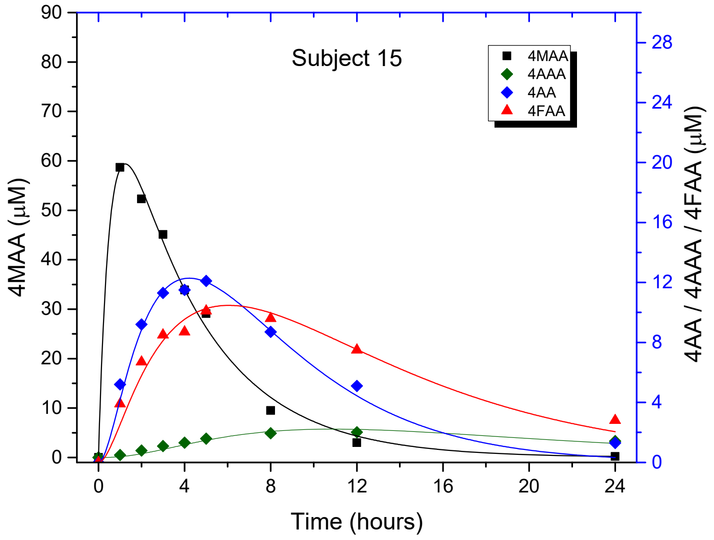

Subject 15
4MAA
4AAA
4AA
4FAA
4MAA (µM)
4AA / 4AAA / 4FAA (µM)
Time (hours)

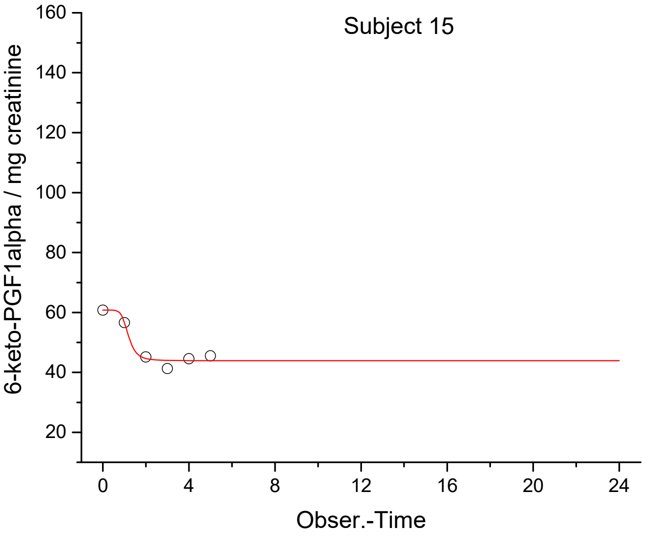

Subject 15
6-keto-PGF1alpha / mg creatinine
Obser.-Time

Individual pharmacokinetic and pharmacodynamic profiles Naproxen

Subject 2

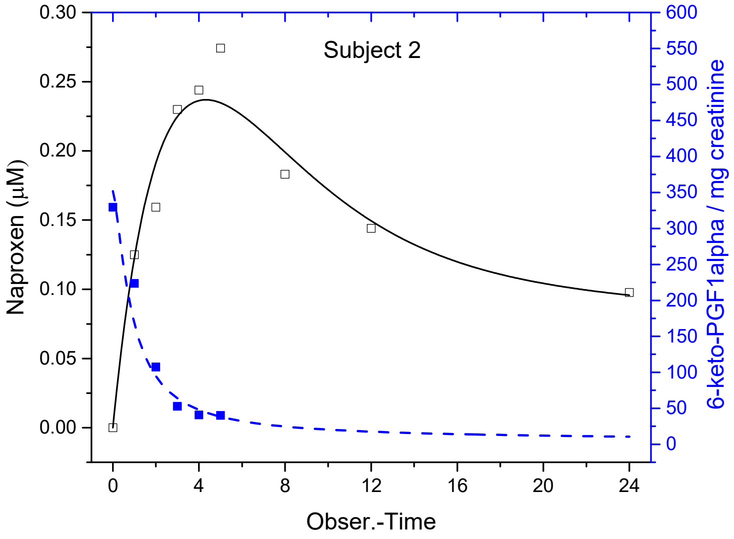

Units for pharmacodynamic effect are uinary exretion of 6-keto-PGF1α in pg/mg creatinine

Subject 3

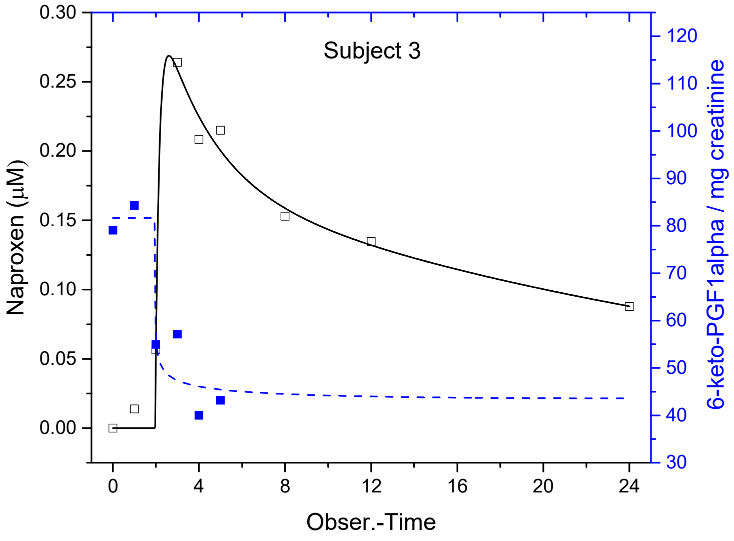

Subject 7

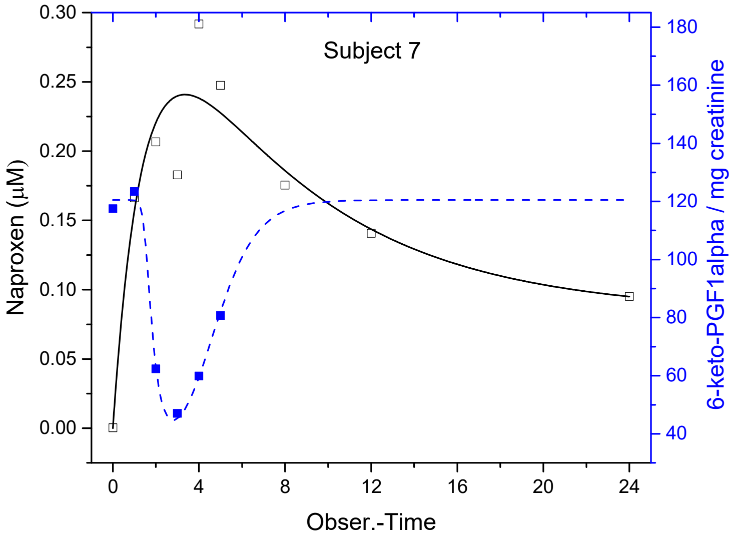

Subject 8

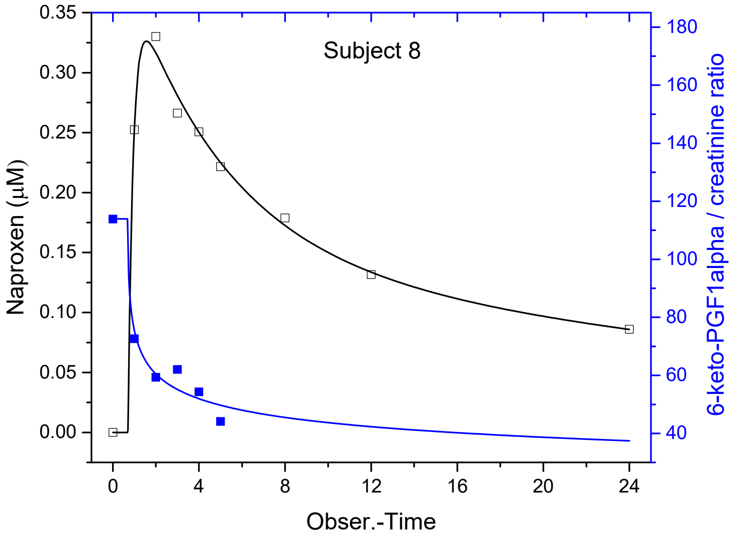

Subject 12

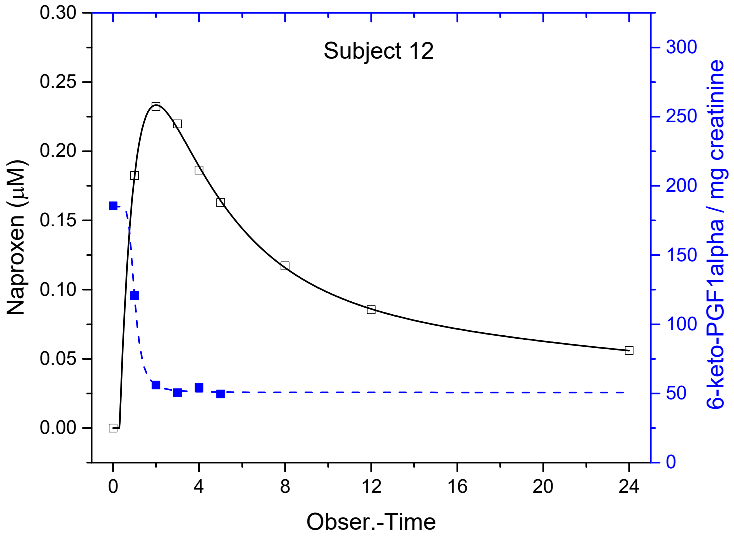

Subject 14

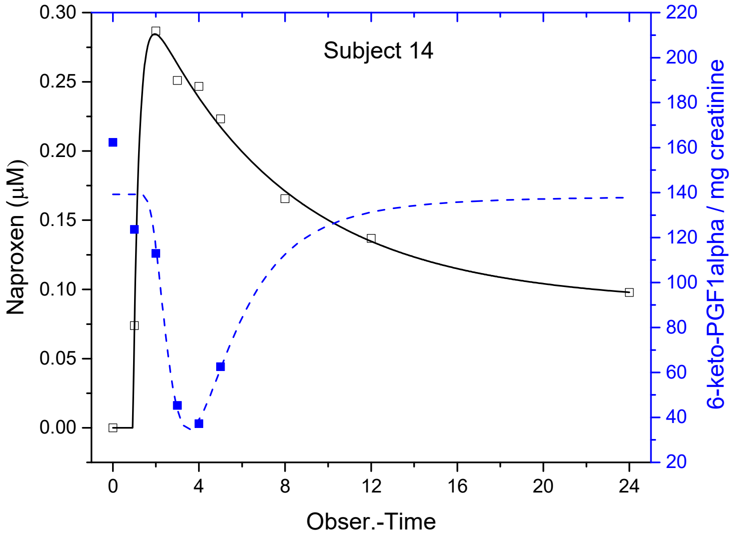

Subject 16

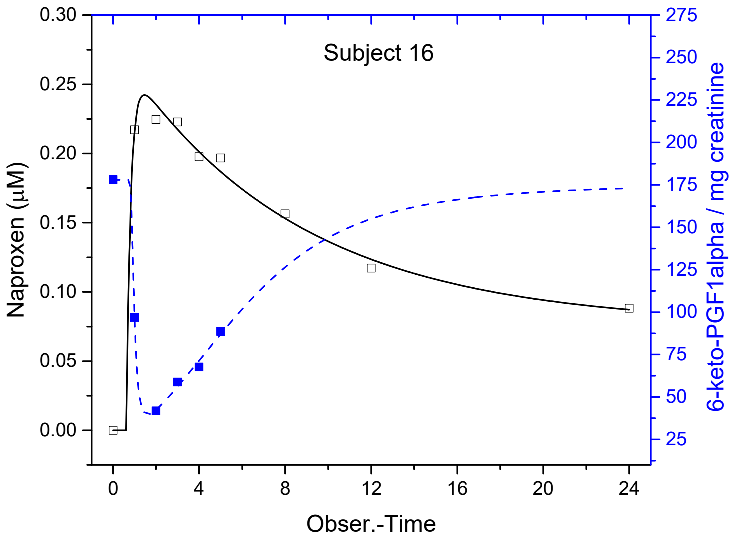

Subject 16
Naproxen (µM)
6-keto-PGF1alpha / mg creatinine
Obser.-Time
0.30
0.25
0.20
0.15
0.10
0.05
0.00
275
250
225
200
175
150
125
100
75
50
25
0
4
8
12
16
20
24
